# Versatile Method for Preparing Two-Dimensional Metal Dihalides

**DOI:** 10.1021/acsnano.4c04397

**Published:** 2024-08-06

**Authors:** Rongrong Qi, Yi You, Magdalena Grzeszczyk, Hiran Jyothilal, Achintya Bera, Jude Laverock, Noel Natera-Cordero, Pengru Huang, Gwang-Hyeon Nam, Vasyl G. Kravets, Daniel Burrow, Jesus Carlos Toscano Figueroa, Yi Wei Ho, Neil A. Fox, Alexander N. Grigorenko, Ivan J. Vera-Marun, Ashok Keerthi, Maciej Koperski, Boya Radha

**Affiliations:** † Department of Physics & Astronomy, University of Manchester, Manchester M13 9PL, U.K.; ‡ National Graphene Institute, University of Manchester, Manchester M13 9PL, U.K.; § Department of Materials Science and Engineering, National University of Singapore, Singapore 117575, Singapore; ∥ Institute for Functional Intelligent Materials, National University of Singapore, Singapore 117544, Singapore; ⊥ Photon Science Institute, University of Manchester, Manchester M13 9PL, U.K.; # School of Chemistry, University of Bristol, Cantocks Close, Bristol BS8 1TS, U.K.; ∇ Department of Physics, National University of Singapore, Singapore 117542, Singapore; ○ Department of Chemistry, University of Manchester, Manchester M13 9PL, U.K.

**Keywords:** two-dimensional materials, metal dihalides, mechanical exfoliation, solvent-assisted recrystallization, photoemission, Raman scattering spectroscopy

## Abstract

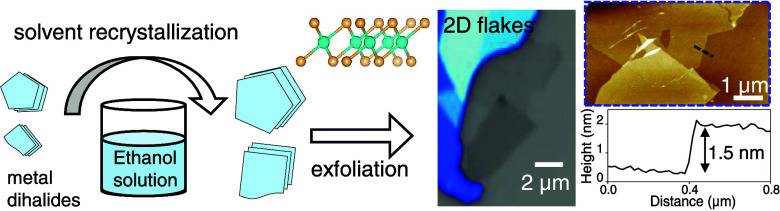

Ever since the ground-breaking isolation of graphene, numerous two-dimensional (2D) materials have emerged with 2D metal dihalides gaining significant attention due to their intriguing electrical and magnetic properties. In this study, we introduce an innovative approach *via* anhydrous solvent-induced recrystallization of bulk powders to obtain crystals of metal dihalides (MX_2_, with M = Cu, Ni, Co and X = Br, Cl, I), which can be exfoliated to 2D flakes. We demonstrate the effectiveness of our method using CuBr_2_ as an example, which forms large layered crystals. We investigate the structural properties of both the bulk and 2D CuBr_2_ using X-ray diffraction, along with Raman scattering and optical spectroscopy, revealing its quasi-1D chain structure, which translates to distinct emission and scattering characteristics. Furthermore, microultraviolet photoemission spectroscopy and electronic transport reveal the electronic properties of CuBr_2_ flakes, including their valence band structure. We extend our methodology to other metal halides and assess the stability of the metal halide flakes in controlled environments. We show that optical contrast can be used to characterize the flake thicknesses for these materials. Our findings demonstrate the versatility and potential applications of the proposed methodology for preparing and studying 2D metal halide flakes.

Two-dimensional (2D) materials have revolutionized materials science. The ability to tailor the surface, chemical, physical, optical, and optoelectronic properties of 2D materials brings application prospects in electronics, energy conversion devices, water purification, and catalysts.^1−3^ The search for 2D materials is an ongoing research direction,^4^ with several 2D families discovered such as metal trihalides with intriguing low-dimensional physics.^5−11^ For instance, the chromium triiodide (CrI_3_) monolayer exhibits an intrinsic ferromagnetic order leading to 2D magnetism.^5^ Further studies on CrI_3_ proposed several ways of tailoring its magnetic property by electrical control, electrostatic doping, and twisting to realize the fabrication of functional devices.^11−13^ Several other metal trihalides also show interesting magnetic properties, e.g., bilayer CrCl_3_ exhibits only in-plane magnetic moments with a little out-of-plane magnetic moment. Few-layered CrBr_3_ is reported as an insulator at room temperature, and at low temperatures (<Curie temperature, *T*_C_), it displays magnon-assisted tunneling^14^ and optical spin pumping^15^ and hosts topological spin textures.^16^

There is increasing research on the metal dihalide family—a group of layered materials that have excellent electrical and magnetic characteristics that are predicted theoretically to be sustained down to the 2D limit.^17,18^ Many of the bulk metal dihalides are magnetic.^19−21^ For example, FeCl_2_ is antiferromagnetic in bulk,^22^ whereas a single layer of FeCl_2_ was predicted in a theoretical study to be ferromagnetic in the ground state.^18,23^ Atomically thin MnI_2_ is antiferromagnetic despite the bulk material being multiferroic.^24^ Though studies imply that it is energetically favorable to obtain monolayers of metal dihalides, so far only a few experimental reports exist on the 2D metal dihalides.^23,25^ Epitaxial growth is a well-known method that has been utilized to synthesize 2D metal dihalides. Examples are FeCl_2_,^23^ FeBr_2,_^26^ and VI_2_,^27^ which have been successfully grown on a Au (111) substrate. Chemical vapor deposition is another proven route to make 2D metal dihalides. Jiang *et al*. synthesized the 2D morphologies of FeCl_2_, FeBr_2_, VCl_2_, and VBr_2_ by reducing their trihalides using a nitrogen-filled interconnected glovebox.^28^ In addition, 2D NiBr_2_ was prepared on a Au (111) substrate by sublimating the NiBr_2_ powder in a Knudsen cell.^25^ Current synthesis methods mostly rely on catalysts and/or substrates to induce crystal growth or chemical deposition. On the other hand, to apply exfoliation methods, we need the parent crystals with high purity and optimization of the sublimation temperature for each crystal. Mechanical exfoliation is a simple method to produce 2D materials with high quality, which led to exploration of several 2D materials. It is a routine method used by several researchers, albeit being time-consuming in terms of flake search and identification of single- to few-layered flakes.^29,30^ To expand research into metal dihalides, we need robust methods that can yield pure crystals and produce 2D materials with ease by exfoliation.

Often, solvent-induced recrystallization methods are overlooked for preparing layered crystals in 2D materials research. Here, we report that bulk layered crystals prepared by simple ethanol-assisted recrystallization can be mechanically exfoliated to make 2D metal dihalides. We illustrate the exfoliation of 2D metal dihalides such as CuBr_2_, CuCl_2_, CoCl_2_, CoI_2_, NiI_2_, and NiBr_2_, showing the versatility of our methodology to prepare a broad range of compounds. The prepared 2D metal dihalides are atomically smooth and flat with flake sizes of a few tens of microns. Although epitaxial growth and CVD methods give high-purity 2D films, the composition and stoichiometry control of the resulting material could be challenging to achieve and is typically achieved by empirically varying the growth parameters. The proposed solvent-induced recrystallization method is highly promising to obtain atomically smooth and thin flakes of 2D metal dihalides with intact composition and clean surfaces and, importantly, with less resource-intensive methods.

## Results and Discussion

**Figure 1:**
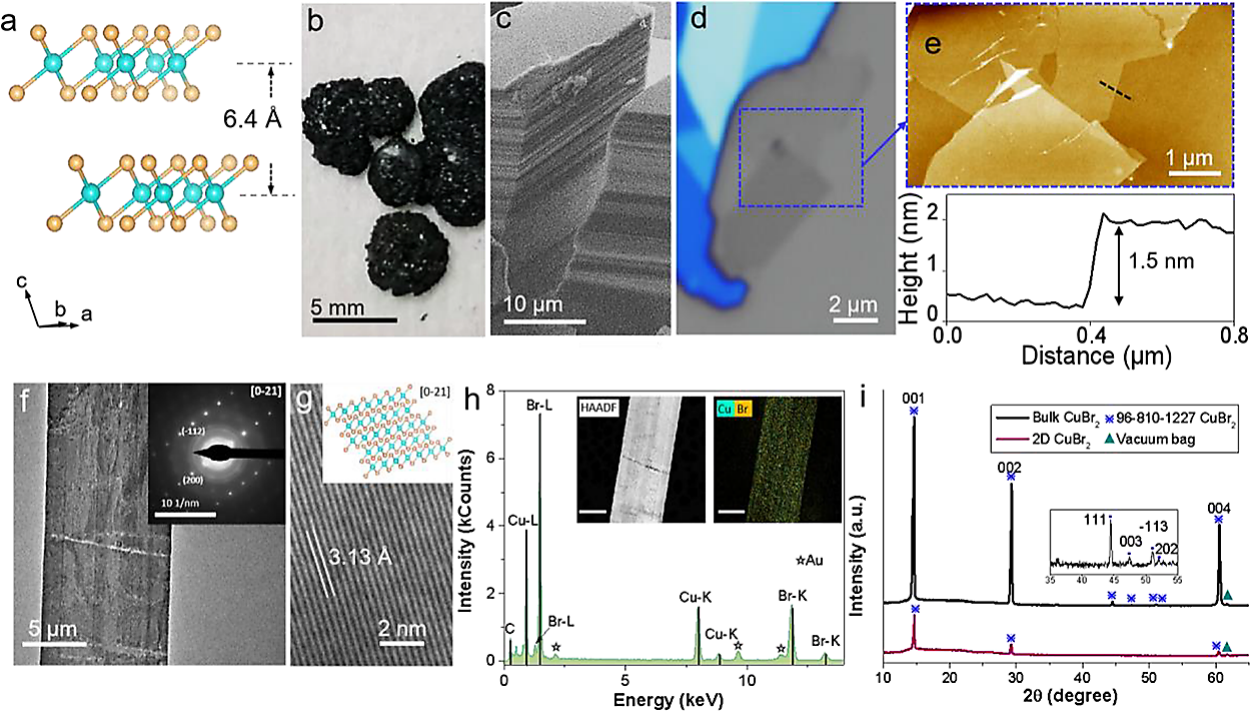
Layered structure and characterization of CuBr_2_. (a) Schematic representation of layered crystal CuBr_2_. The Cu atom is in cyan color, and the Br atom is in orange color. (b) Digital photograph of recrystallized CuBr_2_, and (c) SEM image shows the layered nature of the crystal. (d) Optical image of CuBr_2_ thin flakes. (e) AFM images from a zoomed-in region (blue dashed rectangle) on the optical images. The corresponding height profiles are taken along the black dashed line on the AFM images. (f) TEM image of the CuBr_2_ flake (inset: electron diffraction pattern, see also Supporting Figure S3). (g) High-resolution TEM of the flake with an inset schematic image showing the crystal orientation. (h) EDS spectra of the CuBr_2_ flake with corresponding HAADF-STEM and EDS mapping images in the insets (scale bar, 5 μm). (i) XRD patterns of bulk CuBr_2_ (black) and 2D flakes of CuBr_2_ (red), respectively. The magnified peaks from 35° to 55° (in the inset) from bulk CuBr_2_ are absent in the 2D flakes.

Our method comprises anhydrous solvent-induced recrystallization of bulk powders in a glovebox (details in the Experimental Section), which yields large crystals ranging in the order of >1 mm scale. As an example, we present optical and scanning electron microscopy (SEM) images of the recrystallized CuBr_2_, demonstrating the shiny and layered nature of the crystals (Figure 1b,c, other examples in Figures S1 and S2). With the mechanical exfoliation method using tapes, we prepared 2D flakes from these crystals. The optical images of a thin CuBr_2_ layer with feeble contrast show flakes around ∼1.5 nm thick corresponding to bilayer flakes, as measured by atomic force microscopy (AFM) (Figure 1d,e). A transmission electron microscopy (TEM) image with selected area electron diffraction illustrates the single crystalline structure of the CuBr_2_ flake (Figure 1f and inset). The flake was oriented in the [0 −2 1] crystallographic direction to obtain electron diffraction patterns, which reveal the single crystalline nature with the planes assigned to [2 0 0] and [−1 1 2] planes. A high-resolution TEM image further confirms the crystalline structure of the CuBr_2_ flake with *d*-spacing corresponding to the [2 0 0] crystallographic plane (Figure 1g). High-angle annular dark field scanning transmission electron microscopy (HAADF-STEM) and energy dispersive X-ray spectroscopy (EDS) with mapping (Figure 1h) show the flake composition and atomic fraction ratio of ∼1:2 for Cu and Br, respectively. Structural investigation by X-ray diffraction (XRD) measurements shows that both bulk and 2D CuBr_2_ patterns match well with the XRD data of the nonhydrated CuBr_2_.^31^ Consistent with the XRD pattern of the bulk form (Figure 1i), 2D CuBr_2_ shows diffraction peaks at 14.9°, 28.7°, and 60.6° corresponding to [0 0 1], [0 0 2], and [0 0 4]. These series of parallel planes imply that the obtained 2D flakes are oriented perpendicular to the *c* direction.

**Figure 2:**
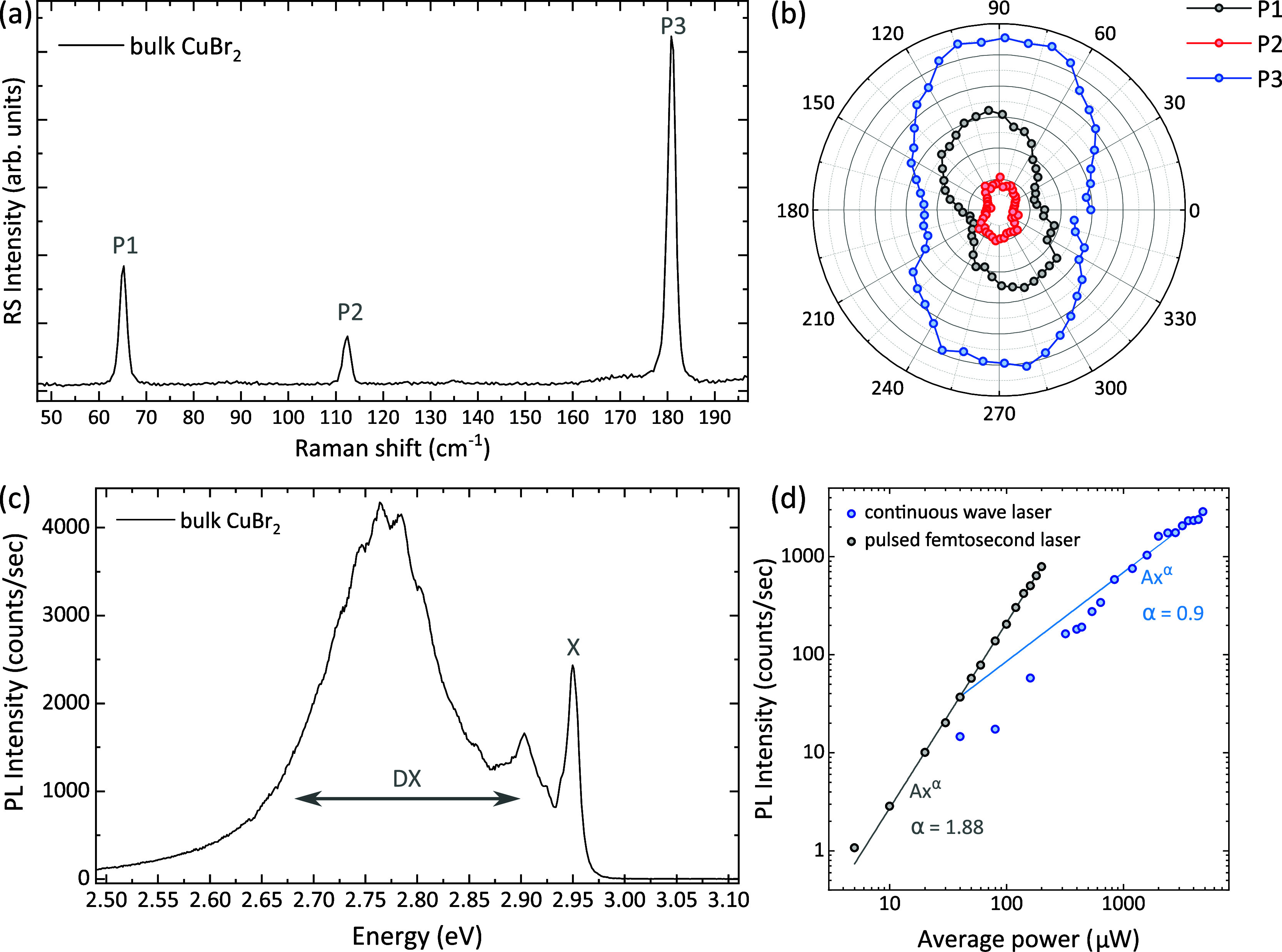
Low-temperature optical spectroscopy of bulk CuBr_2_. (a) Raman spectra obtained at *T* = 5 K with 2.33 eV excitation. Three distinctive features, labeled as P1, P2, and P3, can be observed with energies of 66, 112, and 182 cm^–1^ respectively. (b) Polar plots of the integrated intensities of the P1, P2, and P3 phonon modes. (c). Photoluminescence spectrum of bulk CuBr_2_ under 1.58 eV excitation with a femtosecond laser (75.7 MHz repetition rate). (d) Power dependence measurements of emitted light for continuous wave and pulsed femtosecond laser with excitation energy ∼1.58 eV. The *y* = *Ax*^α^ function has been fitted to both data sets yielding linear and superlinear dependence for continuous wave and pulsed femtosecond laser excitation, respectively. Here, α and *A* are constants, corresponding to the exponent of the power law and the width of the scaling relationship.

One of the intriguing attributes of this family of materials is that they can form a quasi-1D chain structure, in which the metal ions are arranged in a linear chain or zigzag pattern, with the halide ions coordinated around them.^32^ The quasi-1D chain structure can have a significant impact on the optical and electronic properties of materials as it can affect the degree of electron delocalization and the formation of excitonic states, which we investigate using CuBr_2_ with a more thorough characterization of its features. We performed a low-temperature (*T* = 5 K) Raman spectroscopy measurement on bulk CuBr_2_ to study its optical properties. CuBr_2_ belongs to the monoclinic space group C12/m1, which means that its structure is characterized by a unit cell with three atoms. There are nine vibrational modes: three acoustic modes and six optical modes.^33^ The crystal structure consists of edge-sharing CuBr_4_ squares that form ribbons running along the *b* axis. These ribbons are arranged in layers that are parallel to the *ab* plane of the crystal. The CuBr_4_ squares are distorted, with the Cu atoms occupying an off-center position within the squares. In the Raman scattering spectra (Figure 2a), three distinctive peaks (labeled as P1, P2, and P3) can be observed at energies of 66, 112, and 182 cm^–1^. Those lines correspond to the symmetric and asymmetric stretching modes of the Cu–Br bonds.^33^ There are also weaker bands at higher frequencies (see Figure S4), which correspond to bending and other vibrational modes of the CuBr_4_ squares. A more detailed analysis of the polarization of the three main phonon lines is shown in Figure 2b. Both P2 and P3 modes exhibit a 2-fold symmetry with the same polarization axis, while there is a shift of about 30° for the P1 peak. Given the quasi 1D-chain-like structure present in bulk CuBr_2_, we can assume that the polarization properties of the P2 and P3 lines can indicate the crystallographic orientation of those chains in the crystal, while the lowest energy peak can be linked to the in-plane rocking of the Br atoms in the CuBr_4_ square.^33^ Next, we probed the nature of the band gap on CuBr_2_ by measuring photoluminescence and photoluminescence excitation (Figures 2c and S5), with varying excitation power. CuBr_2_ emission has two main features: free neutral exciton and another broadband emission due to donor–acceptor recombination. The obtained spectrum bears a strong resemblance to the well-known emission of PbI_2_ (another representative of the metal dihalide family) as well as hybrid organic–inorganic metal halide perovskites^34,35^ Similar to these materials, CuBr_2_ emission can be induced via two paths: typical Stoke-type emission with an above band gap excitation and anti-Stokes type, when the energy of the emitted photons is higher than that of the absorbed ones. In the anti-Stokes case, the extra energy that causes upconversion of the photons can be acquired through a variety of mechanisms, ranging from multiphoton absorption, energy transfer, and Auger recombination to phonon absorption.^36−38^ To gain more insight into which process is dominant in our case, we resort to power dependence measurements with both continuous wave and pulsed femtosecond laser excitation of the same energy (∼1.58 eV). As can be seen in Figure 2d, the emission intensity is almost proportional to the square of the excitation intensity, suggesting two-photon absorption as one of the mechanisms driving the anti-Stokes emission. A more complete attribution of the emission mechanism is not trivial. It remains an open question for even better-explored materials like in the case of PbI_2_ or perovskites.^34,35^ The upconverted emission of the bulk crystals is not the only interesting aspect of CuBr_2_. In the exfoliated 2D flakes of CuBr_2_ encapsulated in hBN, we found possible candidates for single photon emitters covering the full spectral range of visible to near-infrared wavelengths (see Figure S6 for emission spectra).

**Figure 3:**
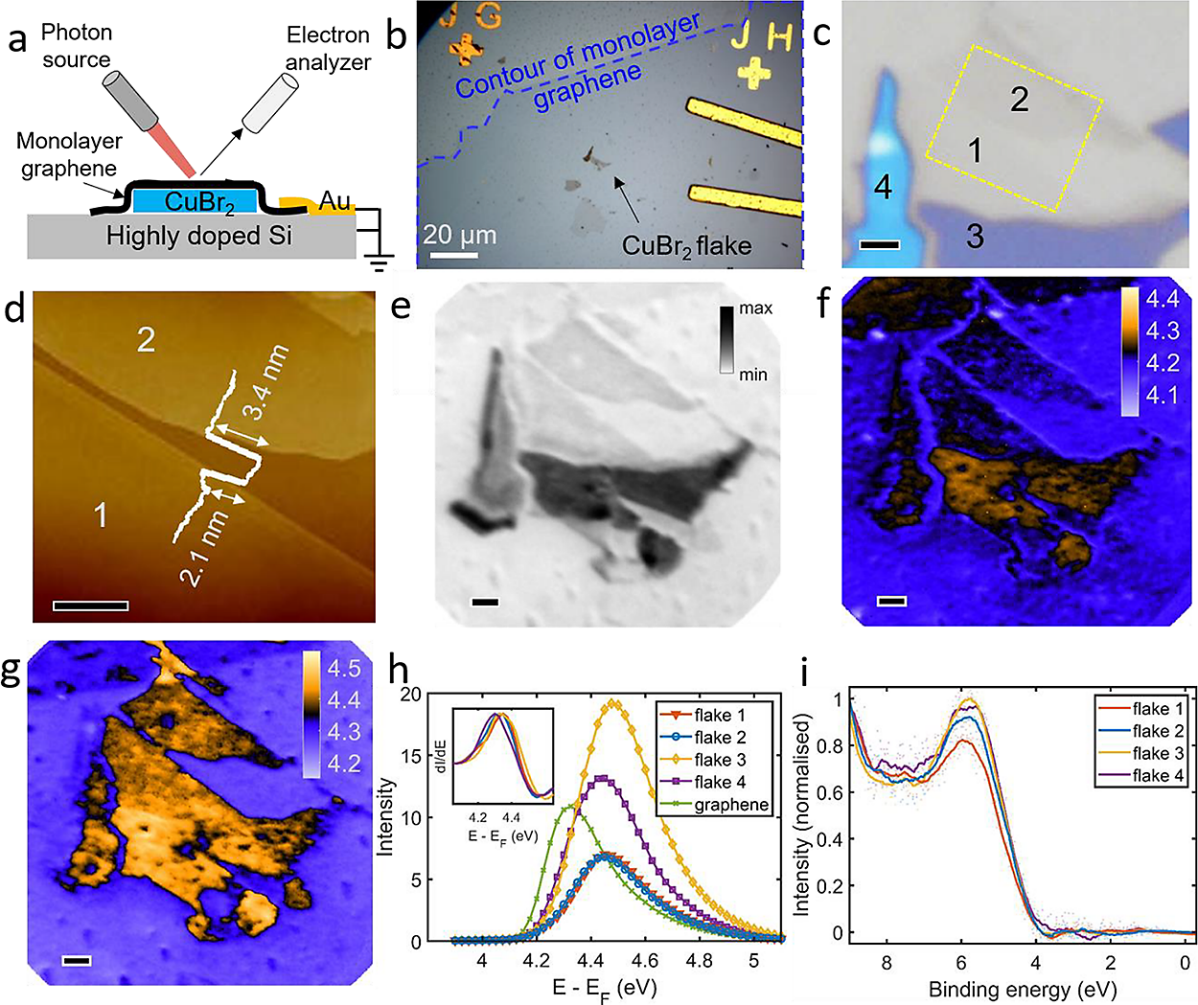
Photoemission spectro-microscopy of 2D CuBr_2_ thin flakes. (a) Schematic diagram of the encapsulated CuBr_2_ with grounding electrodes used for ultraviolet photoemission measurements. The CuBr_2_ 2D flakes were encapsulated in monolayer graphene to prevent degradation as well as to provide grounding. Gold strips were deposited on top of graphene layer to further eliminate the charging issue. The incoming photon source was directed onto 2D flakes, and the analyzer collected the reflected electrons to generate the pattern. (b) Optical image of CuBr_2_ encapsulated with monolayer graphene, with a zoom-in on the CuBr_2_ flake shown in (c). The blue dashed line in (b) is a contour of monolayer graphene. (d) AFM micrograph of the flake in a zoomed-in region (yellow dashed rectangle in image c). (e) Photoelectron microscopy image of the CuBr_2_ flakes encapsulated in graphene, recorded at a photoelectron energy of 4.5 eV. (f) Spatially resolved work function map of the same flakes. The color scale shows the extracted work function in eV. (g) Spatially resolved map of the “cutoff” energy of the secondary electron spectra (color scale in eV). (h) Secondary electron spectra of the flakes and the graphene region. The inset shows the derivative of the spectra near the secondary electron cutoff. (i) UPS of CuBr_2_ flakes recorded with a photon energy of 21.2 eV (He I light source). Solid lines represent smoothed data (using a Savitzky–Golay filter), while raw data are shown as symbols (Figure S9). Scale bars in panels (c)–(g) are 1 μm.

To study the electronic properties of CuBr_2_ flakes using microultraviolet photoemission spectroscopy (micro-UPS), we encapsulated them with graphene, which was contacted with gold electrodes for grounding. Our initial attempts to measure the bare CuBr_2_ flakes (without graphene encapsulation) were unsuccessful due to the low conductivity of the CuBr_2_ flakes (even the thinnest ones), which led to significant charging of the flakes under exposure to ultraviolet light. Therefore, graphene encapsulation was essential to allow electrical draining of the photocurrent, while also protecting the flake. Despite the fact that they originate from a buried layer, there is sufficient transmission of photoelectrons from the CuBr_2_ flakes through graphene to allow meaningful UPS and secondary electron spectra to be acquired, even at extremely surface-sensitive photon energies such as 21.2 eV (He I). When encapsulated, the 2D CuBr_2_ flakes show no sign of degradation during the photoelectron emission microscopy (PEEM)/micro-UPS measurement, which is also confirmed by Raman measurements. Figure 3a depicts a schematic of the micro-UPS measurement for the CuBr_2_ device. A detailed description of the flake preparation and encapsulation process for this sample is given in the Experimental Section and Supporting Information. In brief, we exfoliated the recrystallized CuBr_2_ crystals onto a SiO_2_/Si substrate, and by using poly(methyl methacrylate) (PMMA)/polypropylene carbonate (PPC) dry transfer method, we transferred thin 2D flakes onto a highly doped Si substrate with prepatterned metal markers for navigating the flake during the PEEM/UPS measurement. Then, a graphene monolayer was transferred on top of the flake. Later, the deposition of the gold electrode on top enabled grounding contact, as seen in the optical image (Figure 3b). Underneath the graphene layer, the CuBr_2_ flakes were still visible, although with a faint contrast. A high-magnification optical image and AFM of CuBr_2_ flakes (Figure 3c,d, Supporting Information Figure S7, and Table S1) show flakes that are three-layered (labeled as flake 1) and five-layered (labeled as flake 2).

An energy-filtered PEEM image of the same flakes recorded at a fixed photoelectron energy of 4.5 eV is shown in Figure 3e. At this energy, the contrast is (coincidentally) similar to the optical image, but at other energies, the contrast between different flakes relative to the graphene regions is very high. The spatially resolved work function has been evaluated across this region of interest (Figure 3f) by scanning the photoelectron energy across the secondary electron onset and shows only very small variations of 0.02 eV about a mean work function of 4.21 eV. These small variations are of the order of or smaller than the typical error in extracting the spatially resolved work function and may reflect variations in the local electron density in the vicinity of the graphene overlayer. However, they are too small to explain the contrast mechanism in PEEM, which instead is due to the different shapes of the secondary electron spectra, illustrated by the secondary electron cutoff image (Figure 3g) and the spectra from specific regions of interest (Figure 3h). This is illustrated more clearly in the inset of Figure 3h, which shows the derivative of each spectrum. The mechanisms for secondary electron generation are complex, but in this case, the differences most likely originate from small differences in the available unoccupied densities of states (conduction band) between flakes. For example, the three-layered flake (which is the thinnest flake we measured) has a secondary electron spectrum that is slightly higher in energy than that of the five-layered flake, consistent with a small upshift in its conduction band energy.

Micro-UPS spectra were acquired on the graphene-encapsulated flakes by scanning the photoelectron energy across the valence band region using a photon energy of 21.2 eV. At these photoelectron energies, the inelastic mean free path of an electron in a typical solid is <1 nm, corresponding to an information depth of ≈2 nm, and so we expect the majority of the signal originates from the graphene overlayer. Figure 3i presents micro-UPS spectra of the flakes with this graphene component removed (see Supporting Information Section 4 and Figures S6–S9 for details). As can be seen in Figure 3i, the leading edges of the valence band of the thinner flakes (flakes 1 and 2) are slightly deeper in energy than those of the more bulk-like flakes (flakes 3 and 4), and this downshift is much more marked (∼150 meV) for the thinnest flake 1. These results suggest that the band gap of CuBr_2_ is wider for few-layered flakes.

**Figure 4:**
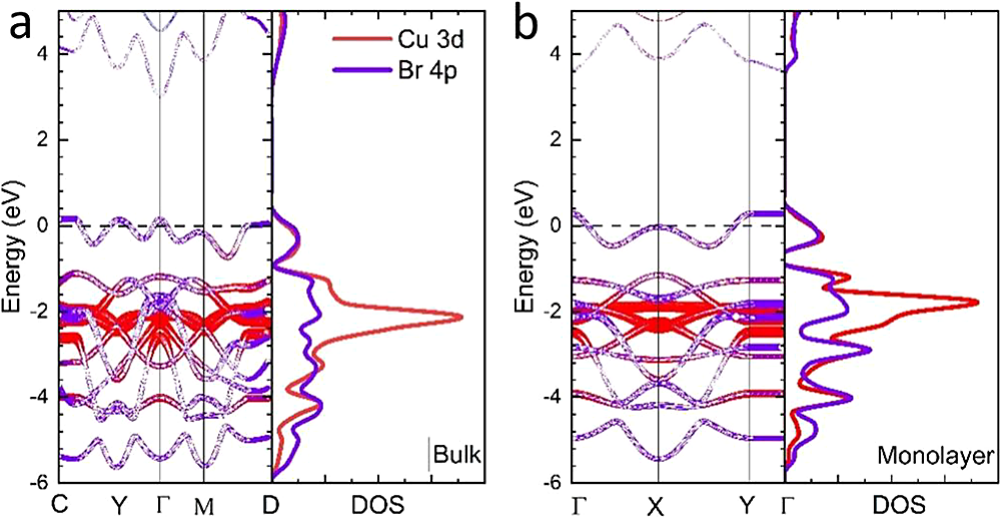
Simulated band structure of bulk and 2D CuBr_2_ via DFT. Electronic band structure and density of states (DOS) of (a) bulk and (b) monolayer CuBr_2_. Both the band structure and DOS are orbital projected to show the contribution of atomic orbitals of Cu and Br.

To understand the electronic structure of CuBr_2_ further, we conducted density functional theory (DFT) calculations to simulate the element-projected electronic band structure (Figure 4) and density of states of bulk and monolayer CuBr_2_. A band gap of ∼3.0 eV was observed. Considering the well-known band gap underestimation in PBE DFT calculations, our results are consistent with the UPS measurement. The valence band edge is contributed mainly by Br 4p states, and the deeper valence sub-band is contributed predominantly by Cu 3d states. Comparing the electronic structure of bulk and monolayer CuBr_2_, one can observe that the band gap of the monolayer is slightly larger than that of the bulk, which is consistent with the spectroscopy results. For deeper insights into the electronic characteristics of CuBr_2_, we constructed a van der Waals heterostructure with a CuBr_2_ flake encapsulated between two graphene flakes on a Si/SiO_2_ substrate (Figure S10). The Dirac curves showed significant hole doping in graphene on CuBr_2_ compared to graphene on SiO_2_ (Figure S10, Table S2). Vertical transport through CuBr_2_ revealed an initial insulating state, followed by a dielectric breakdown and increased conduction. The nonlinear *I*–*V* curves indicated Fowler–Nordheim tunneling, and barrier height estimations were around 0.51 and 1.10 eV (Figure S9, panel e). These findings align with DFT calculations, indicating a larger band gap for few-layered CuBr_2_.

Overall, the comparative analysis of the PL, UPS, and tunneling spectroscopy points toward CuBr_2_ being a large band gap system, which is electrically insulating and hosts strongly localized molecular excitons that follow the Franck–Condon principle revealed by their spectral characteristics. The UPS analysis provides an estimate of the valence band edge to the Fermi level energy separation larger than 4 eV, while the tunneling onsets are indicative of the Fermi level residing 0.5–1 eV below the conduction band edge. These estimations yield a single particle band gap of the order of 5 eV, which would imply an exciton binding energy larger than 2 eV. Consequently, we assigned the highest energy resonance in the PL spectrum at 2.95 eV to the zero-phonon line (labeled as X in Figure 2c), while the lower energy optical response likely originates from the phonon sidebands and/or contributions from defect-bound excitons (labeled collectively as DX in Figure 2c).

**Figure 5:**
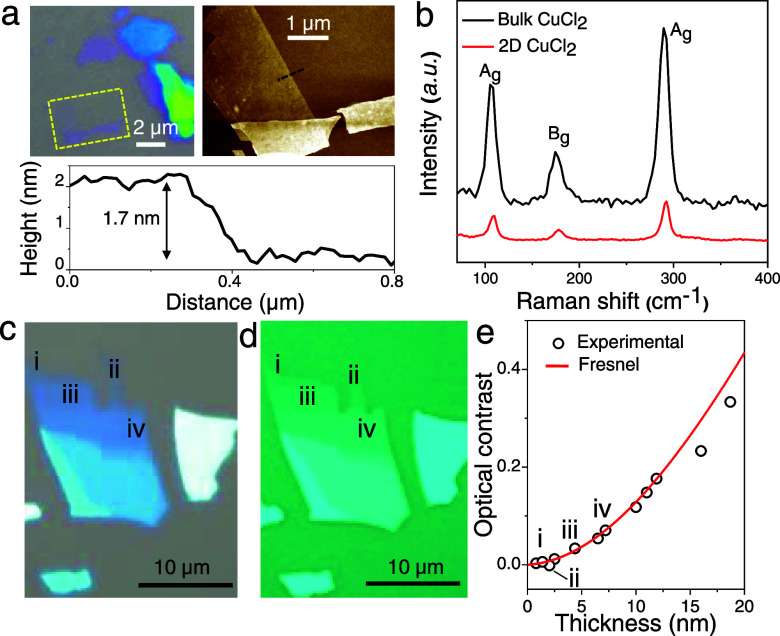
Layered structure of CuCl_2_ and its optical contrast as a function of the layer thickness of a 2D CuCl_2_ flake exfoliated on a 90 nm SiO_2_/Si substrate. (a) Optical image (left) and AFM image (right) from the zoomed-in region (yellow dashed rectangle) on the optical image. The corresponding height profile (below) was taken along the black dashed line in the AFM images. (b) Raman spectra of both bulk and 2D CuCl_2_. High-magnification optical image of CuCl_2_ (c) under white light and (d) under a light filter that blocks wavelengths below 500 nm. (e) Comparison between the experimental data of the optical contrast against the layer thickness obtained by AFM imaging; the red line shows the data fit using the Fresnel function (see Supporting Section 8). The labeled dots with i–iv in panel (e) correspond to the flakes in the optical images (c) and (d).

We apply our methodology of preparing 2D flakes of further metal halides, namely CuBr_2,_ CuCl_2_, CoCl_2_, CoI_2_, NiBr_2_, and NiI_2_ (Supporting Section 6, Figures S11–S16). The optical images of exfoliated trilayer 2D CoCl_2_, four-layer 2D CoI_2_, and four-layer 2D NiBr_2_ are shown in Figure S12. The Raman spectrum of 2D CuCl_2_ matches well with that of the bulk with sharp peaks at 110, 180, and 290 cm^–1^ corresponding to the Raman modes of A_g_, B_g_, and A_g_, respectively (Figure 5b). With CuCl_2_, we explore optical contrast as a rapid identification method for flake thickness,^5^ which is highly advantageous for those 2D materials that are sensitive to exposure to air and/or water. By keeping track of the CuCl_2_ optical contrast and correlating their thickness extracted from AFM, we constructed the calibration curve shown in Figure 5 (further details in the Experimental Section and Figure S14). An example image of the CuCl_2_ flake with various thicknesses is shown in Figure 5a, where the thinnest region is ∼1.7 nm, which is a trilayer of CuCl_2_. From the AFM images, it is evident that these 2D flakes have a smooth surface. The best contrast for CuCl_2_ was observed on a 90 nm-thin SiO_2_/Si substrate when viewed through a 500 nm long-pass filter (Figures 5d and S17). The calibration curve of optical contrast to the measured flake thickness was also computed using a model based on the Fresnel equation (details in Supporting Information Section 8), and the predicted trend agrees well with the experimentally obtained optical contrast (Figure 5e).

**Figure 6:**
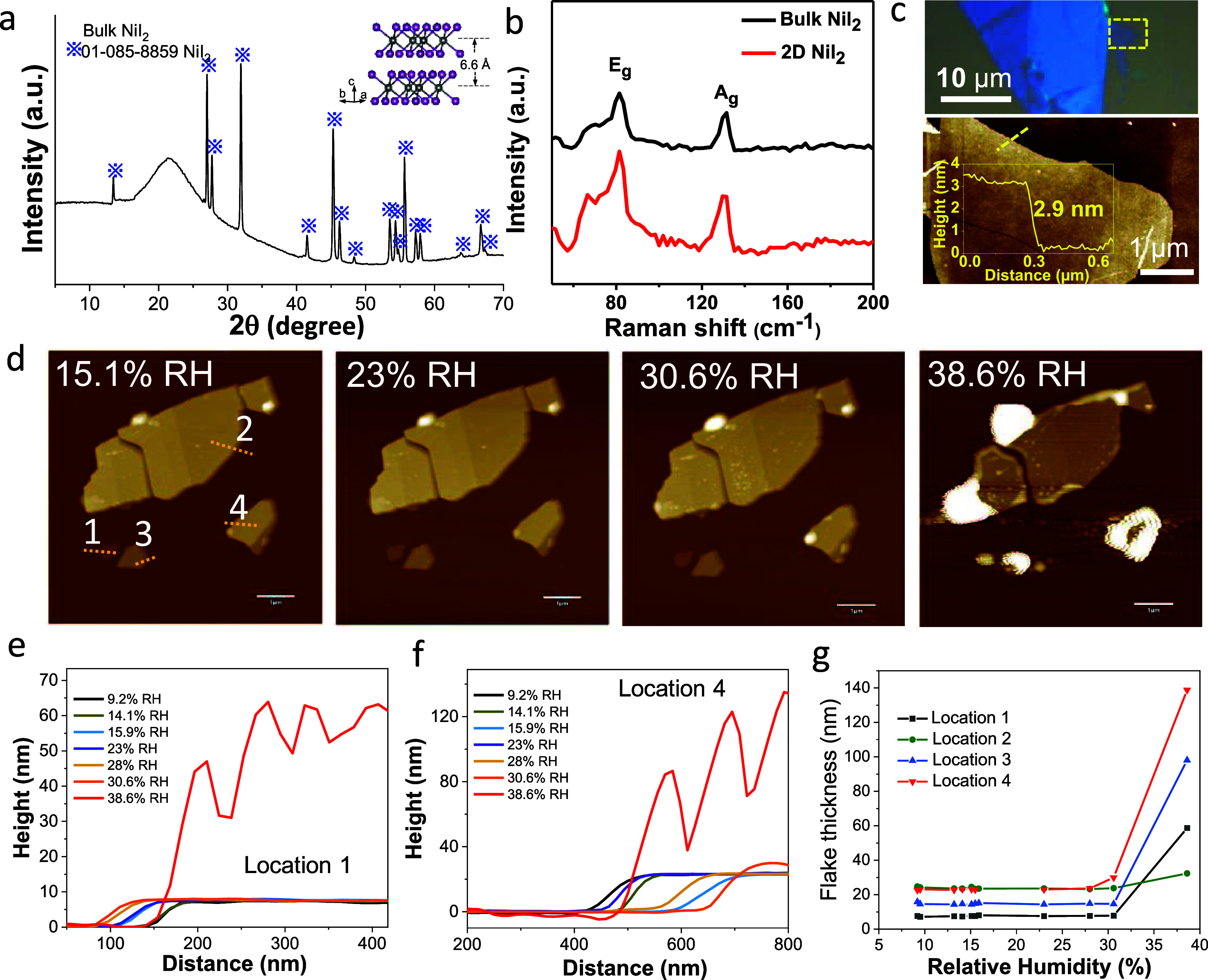
Layered structure and characterization of NiI_2_. (a) XRD patterns of bulk NiI_2_. Inset: schematic layered crystal structures of NiI_2_. The Ni atom is in gray color, and the I atom is in purple color. (b) Raman spectra of both bulk (black color) and thin flake (red color) of NiI_2_. (c) Optical (top) and AFM (bottom) images from the dashed yellow rectangle shown on the optical image. The AFM superimposed height profile was taken on the direction of the yellow line. (d) AFM images showing the hydration progression of freshly exfoliated NiI_2_ flakes at 4 different humidity levels (full range of RH in Figure S16). Scale bar: 1 μm. Height profiles at (e) location '1' and (f) location '4' (as indicated in the image d) with increasing RH. (g) Flake thickness vs RH for different places of the flake as marked in (d).

To examine the metal halide flakes’ physical and chemical stability, we conducted a systematic test by exfoliating the flakes in a controlled environment (glovebox) and in the air (Figure S13). To ensure all the flakes and crystals remained in the anhydrous state and to avoid any environmentally driven degradation, we enclosed the samples inside a hermetic cell within an inert environment while analyzing them using Raman spectroscopy (Figure S13). On the 2D flakes exposed to the ambient condition after exfoliation, we can visualize water droplet formation in ambient conditions, likely due to the highly hygroscopic nature of the halides. The Raman spectra and AFM of the flakes show the ambient air- and water-induced deterioration of the flakes (Figures S14–S16). Furthermore, while exposed to ambient air, we monitor the degradation with 2D NiI_2_ flakes as an example, using continuous imaging by AFM. Bulk NiI_2_ belongs to the *R**m* space group, with *a* = 3.9 Å, *b* = 3.9 Å, *c* = 19.6 Å, and β = 90° (Figure 6a, XRD data), and a typical exfoliated 2D NiI_2_ (four-layer) is shown in Figure 6c. The Raman peaks of bulk and 2D NiI_2_ E_g_ and A_g_ are positioned at 82 and 130 cm^–1^, respectively (Figure 6b). The hydration progression via AFM of the thin NiI_2_ flakes is shown in Figure 6d–f. In a glovebox, the samples were loaded inside a sealed holder that enables in situ AFM imaging in an inert atmosphere. The imaging was done under a nitrogen atmosphere, and the relative humidity (RH) was controlled by purging a controlled amount of ambient air into the sealed chamber. We initiated the imaging at around 9.2% humidity level (Figure 6d), and images of the flakes at different RHs of 15.1 to 38.5% are presented (Figure S16). At >30% RH, the surface is increasingly rough with white blisters. At 38.6% RH, the surface increasingly degrades, and the thickness of the flakes also increases. Below 30% RH, the thickness does not change much (height profiles across locations “1” and “4” shown in Figure 6f), but above this, the thickness of the flake increases sharply. Several areas of the flakes marked “1–4” in Figure 6d show a similar trend in that the thickness of all the flakes did not change greatly when the humidity was below 30% RH. Such degradation can be prevented by encapsulation with stable and chemically inert 2D materials (such as hexagonal boron nitride or graphene) or by preserving the layers through immersion in specific acidic solutions.^39^

## Conclusions

In conclusion, we have established a simple and robust method for preparing bulk crystals of metal dihalides by solvent-induced recrystallization and exfoliating them into 2D flakes. The bulk crystals are layered and highly crystalline, as evident from the TEM and XRD measurements. The obtained 2D flakes exhibit smooth surfaces, atomically thin, and of the size of a few tens of micrometers, allowing spectroscopic characterization. Several metal dihalides (Cu, Co, and Ni families) have been produced with our method, demonstrating the versatility of our approach. CuBr_2_, which has been extensively investigated, has a quasi-1D chain structure and has broadband emission from donor–acceptor recombination. We further characterize the electronic structure using micro-UPS, where CuBr_2_ exhibited a uniform work function on the flakes overall. We show the thickness–optical contrast map of the 2D flakes as a guide to determine the metal halide flake thickness. The DFT calculations and electron-tunneling experiments confirm the wide band gap. Our results here show simple methods of producing bulk layered crystals for exfoliation to 2D flakes, which can be expanded to other types of 2D materials such as iron dihalides (FeCl_2_ and FeI_2_), manganese dihalides (MnBr_2_), perovskites, and oxides.

## Methods/Experimental Section

### Solvent-Assisted Recrystallization of Bulk Metal Dihalides

In this study, we presented the 2D flakes of six metal halides (CuBr_2_, CuCl_2_, CoCl_2_, CoI_2_, NiI_2_, and NiBr_2_). In brief, each anhydrous bulk material of CuBr_2_ (99%, Acros Organics), CuCl_2_ (99%, Sigma-Aldrich), CoCl_2_ (99%, Alfa Aesar), CoI_2_ (99%, Alfa Aesar), NiI_2_ (99%, Alfa Aesar), and NiBr_2_ (99%, Alfa Aesar) was transferred individually to a 5 mL beaker and dissolved in anhydrous ethanol (99%) solution until saturation. Let us describe the recrystallization procedure using CuBr_2_ as an example. First, ∼0.1 mg of the bulk solid was dissolved in ∼0.3 mL of ethanol. The solution was placed in a controlled environment of 25 °C and <0.5 ppm RH% inside a glovebox. Then, we allowed a gradual evaporation of anhydrous ethanol solvent in the glovebox; what remained in the beaker was a crystalline solid as shown in Figures 1 and S1, which is the anhydrous solid. In order to confirm the crystallographic phase of this material, we placed a small amount of these solids into a quartz glass capillary tube of 0.5 mm diameter and sealed it with plasticine to perform powder-XRD measurement, using a dual-wavelength Rigaku FR-X rotating anode CuKα diffractometer (λ = 1.54146 Å) radiation, equipped with an AFC-11 4 circle kappa goniometer, VariMAXTM microfocus optics, a Hypix-6000HE detector, and an Oxford Cryosystems 800 plus nitrogen flow gas system, at a temperature of 298 K. The beam divergence was set to 1.0 mR. Data were collected and reduced using CrysAlisPro v42. The XRD analysis showed the unit cell parameters: *a* = 7.2 Å, *b* = 3.5 Å, *c* = 7.0 Å, and β = 119.6°, belonging to the C12/m1 space group. The flake exfoliation was performed using the so-called Scotch tape method onto Si/SiO_2_ substrates. The substrates containing the exfoliated flakes of several micrometers in size were stored in a vacuum bag for protection against degradation in ambient conditions. Besides powder-XRD, standard XRD was used for the 2D CuBr_2_ flakes. The diffraction pattern obtained with a Rigaku XRD diffractometer with a 200 μm beam spot revealed diffraction patterns corresponding to the [0 0 1], [0 0 2], and [0 0 4] planes, with positions identical to that of the bulk crystals. We performed Raman spectroscopy (Horiba Xplora+) using a 532 nm laser source, with the samples mounted in the aforementioned hermetic cell, which retains the inert atmosphere of a glovebox (further details of the cell in Supporting Information Figure S12). Through this recrystallization process, the bulk crystal aggregated and became larger than the commercially purchased powders, which led to large 2D flakes upon exfoliation.

### Flake Preparation

Mechanical exfoliation of metal halide crystals was done in a glovebox (<0.5 ppm of water and oxygen) using adhesive tapes to repeatedly delaminate the obtained bulk crystals, followed by pressing against a SiO_2_/Si substrate to further decrease its thickness down to a few atomic layers. We note that heating the substrate to around 130 °C prior to exfoliation helps to exfoliate larger and thinner flakes. The yield of monolayer and bilayer flakes (few microns in dimension) would be less than 1% of total flakes on a typical substrate (290 nm SiO_2_/Si wafer). The majority of exfoliated flakes are >30 nm, and around 5–10% of flakes are below 5 nm-thick flakes.

### Optical Microscopy Measurement

Optical microscopy images were captured by using a microscope (Nikon) housed in a glovebox to prevent decomposition of the metal halide flakes caused by moisture and oxygen. The quantitative contrast data were collected from the NIS-Elements BR software on the microscope, with the flakes captured under a 100× objective using a 500 nm long-pass optical filter, which blocks the light less than 500 nm. The reflection intensity values from the filter were extracted from the flake and substrate to obtain the difference in intensity (further details in Supporting Information Section 8). The experimental optical contrast of the flake with respect to the substrate was noted following the Michelson contrast equation.^5^ The flakes were placed in the hermetic cell in a glovebox and then transferred to an AFM stage to qualitatively measure the thickness. The experimental data were then plotted and fit using the Fresnel equation by using the refractive index and extinction coefficient acquired from the ellipsometry instrument on the bulk CuCl_2_ (see Figure S14c). There is a good match between the simulation and experimental results. Thus, optical contrast can be used as an indicator for the thickness of the CuCl_2_ flake (Figure 5e).

### Stability of the 2D Flakes

Metal halide flakes can disintegrate in just a few seconds when exposed to ambient conditions because of their hydration. To study their stability, we mounted the substrates in a hermetic cell inside a glovebox and used Raman spectroscopy as a quick check.^40^ When sealed in the hermetic cell, the samples can survive while performing the Raman measurements, without any noticeable H_2_O peak at 3400 cm^–1^ and retaining the characteristic peaks of halides before 400 cm^–1^. The Oxford Cypher ES Environmental atomic force microscope (AFM) was used to monitor the continuous hydration of the flakes. The samples were mounted in a customized glovebox transfer cell that enables AFM imaging while maintaining an inert atmosphere. Once the sample holder was loaded inside the AFM system, we continuously passed dry N_2_ gas to maintain the desired RH level. Controlled release of air was done to hydrate the flakes using a flow meter while imaging with AFM.

### Transmission Electron Microscopy (TEM)

TEM imaging was carried out using FEI Tecnai G2 20 operated at 200 kV, and HAADF-STEM/EDX spectrum image data were acquired using a Thermo Fisher Scientific Talos F200X microscope operated at a 200 kV accelerating voltage. The single crystalline flake of CuBr_2_ was oriented by following Kikuchi bands in the shortest time to minimize beam damage, which helped in acquiring SAED patterns and HRTEM images. HAADF-STEM data were acquired with a probe current of 260 pA, and the same nanoprobe was used for EDX mapping/spectra with a pixel dwell time of 20 μs. The thin flake of CuBr_2_ was transferred to a Au grid (Au TEM grids with a holey carbon support film) for measurement via PDMS in the glovebox.

### Photoelectron Emission Microscopy (PEEM)

PEEM measurements were performed using a Focus/Scienta Omicron NanoESCA II instrument in the Bristol Ultraquiet NanoESCA Laboratory (BrUNEL), which is equipped with a broadband mercury lamp for imaging and a monochromatic helium discharge lamp (He I, 21.2 eV) for spectroscopy. All measurements were performed under ultrahigh vacuum at a base pressure of ≈2 × 10^–11^ mbar. The electron analyzer was set to an energy resolution of 100 meV (50 eV pass energy), and the field of view was ≈18 μm. Work function measurements were performed by scanning the electron analyzer energy near the secondary electron cutoff energy, and the spatially resolved work function was extracted by fitting the spectra under each pixel to a combination of two error functions.^41^ Secondary electrons are generated up to several nm beneath the surface, and although the work function is a property of the surface, the energy spectrum of secondary electrons contains information on the available unoccupied states (e.g., conduction band)^42^ and excitations (e.g., phonons)^43^ of the sensitive volume. In contrast, photoelectrons in the valence band region of a UPS measurement are sensitive to just the top few monolayers at He I energies.

### Microultraviolet Photoemission Spectroscopy (Micro-UPS)

Micro-UPS measurements were performed by scanning the analyzer energy across the valence band, acquiring PEEM images at each step. Long acquisition times of 90 s per energy were used, corresponding to 14 h overall. To minimize sample drift during the acquisition, images were acquired every 3 s, and reference images were regularly recorded to track and correct for sample movement. Raw micro-UPS spectra were subsequently extracted by defining regions of interest for each flake and the graphene overlayer and spatially integrating within these regions. Then, we obtained spectra representative of the CuBr_2_ flakes by subtracting a component due to graphene (see the Supporting Information for details).

### Device Fabrication for PEEM and Micro-UPS Measurements

The 2D CuBr_2_ thin crystal was initially exfoliated first on a SiO_2_/Si substrate, as the oxidation layer provides good optical contrast benefiting the flake searching process. The fabrication procedure is detailed schematically in Figure S7. The 2D flakes were then dry transferred via the PPC/PMMA stamp method onto a highly doped Si substrate containing metal markers of a 100 μm pitch size. These developed markers aid the navigation to the region of interest when conducting the UPS measurement. Subsequently, a single layer of graphene was uniformly placed above the 2D CuBr_2_ flake through a PMMA-assisted dry transfer method. This graphene layer eliminates the charging by good grounding of the flake. Note that we conducted Raman spectroscopy before and after the graphene dry transfer to confirm that the CuBr_2_ flake remains chemically and physically stable with no degradation. Later, metal strips (5 nm Cr/60 nm Au) were made via photolithography and electron beam deposition above the monolayer graphene away from the CuBr_2_ flakes.

Samples for PEEM and UPS measurements were degassed under ultrahigh vacuum at 350 °C for 1.5 h to remove surface absorbates before transferring to the PEEM chamber. Angle-resolved photoemission spectroscopy measurements of the graphene overlayer exhibited characteristic graphene Dirac cones at the K points of the Brillouin zone, confirming the clean and well-ordered surface of graphene in these devices. Initial measurements of samples that had not been contacted with graphene suffered from substantial charging during photoemission measurements due to the nonconducting nature of CuBr_2_.

### Raman and PL Spectroscopy

PL and Raman scattering experiments were measured in a backscattering microscopic configuration in a dry cryogenic system at 4.2 K. Samples were mounted on piezoelectric stages, allowing *x*–*y*–*z* positioning. The laser light was focused using a lens with a numerical aperture of 0.82, yielding a spot of approximately 1 μm in diameter. Light emitted from the sample was collimated by the same objective and scattered by a 0.75 m spectrometer equipped with 150 and 1800 lines/mm grating and a charge-coupled device camera.

For the Raman and PL measurements, all exfoliation and transfer steps of flakes were carried out in a glovebox by dry transfer techniques. The 2D CuBr_2_ thin flakes were encapsulated between hBN flakes. The CuBr_2_ flakes were directly exfoliated on PDMS and then transferred onto bottom hBN (thickness, ∼15 nm) on a SiO_2_/Si substrate with a 5 mm × 5 mm size. To locate them easily, the selected flakes were placed on a corner within a 1 mm × 1 mm area, at room temperature. To protect the sensitive CuBr_2_ flakes, the top hBN (with a thickness of <10 nm) was picked up by the PDMS/PPC stack and then dropped off to seal the CuBr_2_ flakes.

### DFT Calculations

Our calculations were based on DFT using the PBE functional as implemented in the Vienna Ab initio Simulation Package. The interaction between the valence electrons and ionic cores was described within the projector-augmented approach with a plane-wave energy cutoff of 500 eV. Spin polarization was included for all of the calculations. The Brillouin zone was sampled using a (31 × 31 × 31) Monkhorst–Pack grid. A 20 Å vacuum space was used to avoid the interaction between neighboring layers for the monolayer calculation. In the structural energy minimization, the atomic coordinates were allowed to relax until the forces on all of the atoms were less than 0.01 eV/Å. The energy tolerance was 106 eV.

## Supporting Information Available

Images of recrystallized and bulk metal halides; simulated and experimental electron diffraction patterns; additional Raman and photoluminescence characterization of CuBr_2_; micro-UPS sample preparation details and additional spectra; electrical transport measurements of CuBr_2_; XRD, optical and AFM images, and Raman spectra of other metal halides; stability measurements of metal halides; and modeling of optical contrast (PDF)
