# Supplementary material for: Versatile Method for Preparing Two-Dimensional Metal Dihalides
Source: ACS Nano. 2024 Aug 6;18(33):22034–44. doi: 10.1021/acsnano.4c04397 (PMC11342368; doi:10.1021/acsnano.4c04397)
Supplement: Supplementary file 1 — nn4c04397_si_001.pdf [file nn4c04397_si_001.pdf]

Supporting information for

Versatile Method for Preparing Two-Dimensional Metal Dihalides

Rongrong Qi[1,2,#], Yi You[1,2,#], Magdalena Grzeszczyk[3,4], Hiran Jyothilal,[1,2] Achintya Bera[1,2,5], Jude Laverock[6], Noel Natera-Cordero,[1,2] Pengru Huang[3,4], Gwang-Hyeon Nam[1,2], Vasyl G. Kravets[1], Daniel Burrow[1,2], Jesus Carlos Toscano Figueroa[1], Yi Wei Ho,[4,7] Neil A Fox[6], Alexander N. Grigorenko[1], Ivan J. Vera-Marun[1,2], Ashok Keerthi[2,8], Maciej Koperski[3,4 *], Boya Radha[1,2 *]

[1]Department of Physics & Astronomy, University of Manchester, Manchester M13 9PL, UK

[2] National Graphene Institute, University of Manchester, Manchester M13 9PL, UK

[3]Department of Materials Science and Engineering, National University of Singapore, Singapore 117575, Singapore

[4]Institute for Functional Intelligent Materials, National University of Singapore, Singapore 117544, Singapore

[5]Photon Science Institute, University of Manchester, Manchester M13 9PL, UK

[6]School of Chemistry, University of Bristol, Cantocks Close, Bristol BS8 1TS, UK

[7]Department of Physics, National University of Singapore, Singapore 117542, Singapore

[8]Department of Chemistry, University of Manchester, Manchester M13 9PL, UK

# authors contributed equally

*correspondence to radha.boya@manchester.ac.uk; msemaci@nus.edu.sg

1. Recrystallization of metal dihalides (MX_2, M=Cu, Co, Ni, X=Cl, Br, I)

To prevent the hydration of metal dihalides, we recrystallized them in an inert atmosphere inside a glovebox. The metal halides were dissolved in anhydrous ethanol (99.9%) to form a saturated solution. The solution was then left to evaporate to form crystals of MX_2. The MX_2 crystallites were left in the inert atmosphere to dry for half a day. Fig. S1 shows the digital images of the metal dihalides which are purchased and recrystallized, respectively. Furthermore, scanning electron microscopy (SEM) images reveal the layered structure evidently in the after recrystallization (Fig. S2).

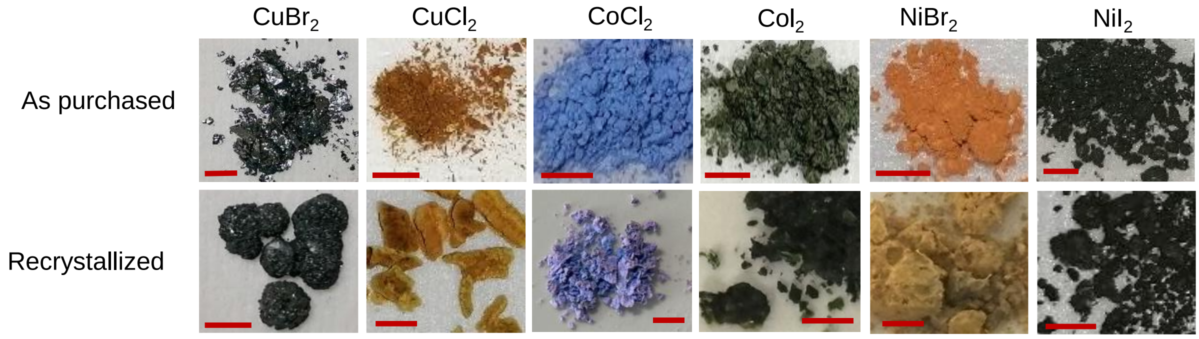

Figure S1. Digital graphs of the as purchased and as recrystallized metal-based (Cu, Co, Ni) bromide, chloride and iodide. Scale bar, 2 mm.

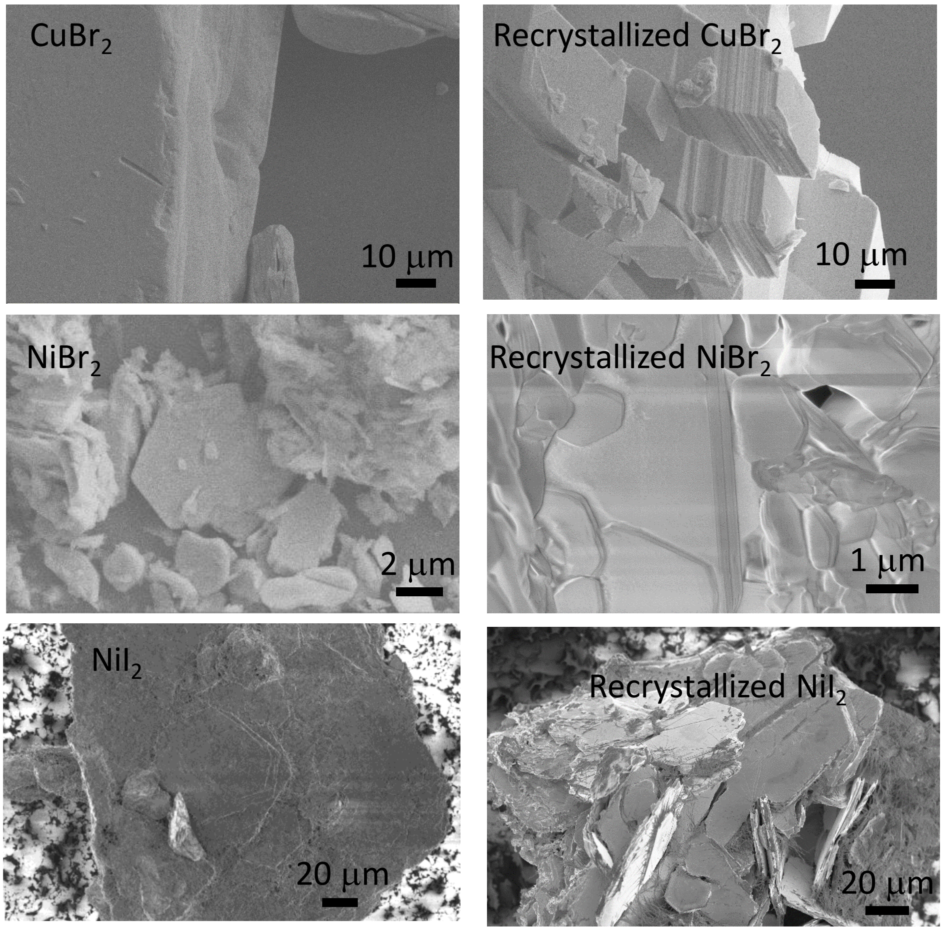

Figure S2. SEM graphs of the as-purchased and as-recrystallized $CuBr_2$, $NiBr_2$ and NiI_2. The faint horizontal lines seen in the $NiBr_2$ and NiI_2 images is due to charging.

2. Electron diffraction

We simulated an electron diffraction pattern using reported CIF file 409450 of $CuBr_2$ in the SingleCrystal software, and this was the basis of our indexing in the experimentally collected diffraction data (Fig. S3a). Upon exposure of more than few minutes to the electron beam, hazy polycrystalline rings were observed (Fig. S3c). This could be attributed to the beam damage caused by the electron beam on the $CuBr_2$ flake. Polycrystalline rings brighten upon prolonged electron beam exposure (Fig. S3 b&c).

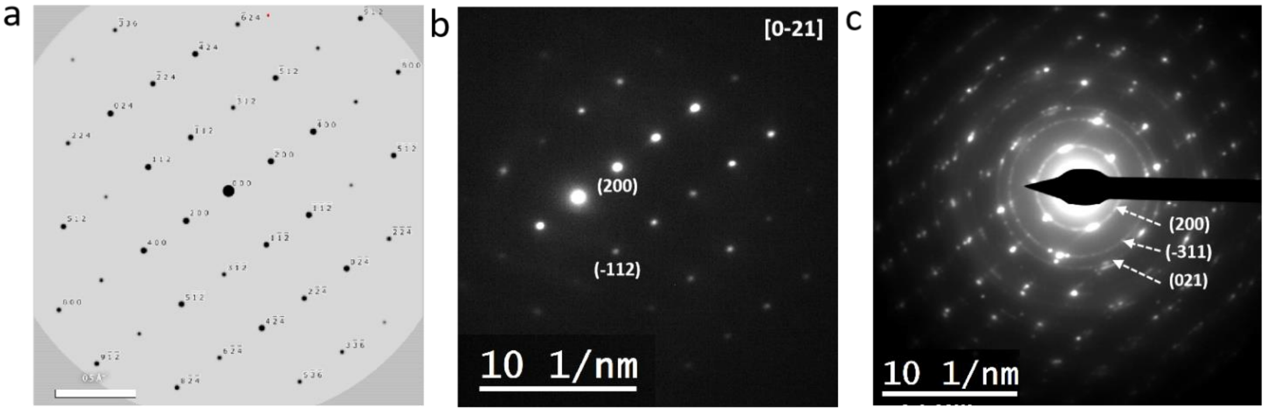

Figure S3. Simulated Electron Diffraction of $CuBr_2$ flake in [0 -2 1] crystallographic orientation using CIF file 409450 from Crystallography Open Database. (b) SAED pattern of $CuBr_2$. (c) SAED pattern after degradation elucidating both single crystalline and polycrystalline patterns.

3. Raman and photoluminescence spectroscopy of $CuBr_2$

In the $CuBr_2$ Raman spectrum, multiple resonances can be recognized over a wider energy range. While the three lowest energy features are assigned to symmetric and asymmetric stretching modes of the Cu-Br bonds the higher energy broad lines are not yet interpreted. Those vibrations are related to spin excitations and are presumably dominated by two-magnon scattering[1]. They can be detected well above Néel temperature T_N, suggesting that the short-range spin correlations are present even at room temperature.

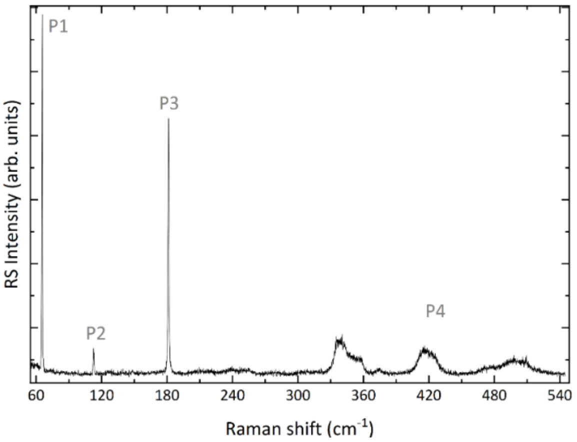

Figure S4. Raman spectra obtained at T = 5 K with 1.58 eV excitation. Beside the three distinctive peaks labelled as P1, P2, and P3 described in the main text additional broad features are visible at higher energies.

The comparative analysis of PL and PLE spectra allows us to identify two resonances, one perfectly matching the energy of the emission of the free exciton, second shifted by ca. 0.2 eV to higher energies (Fig. S5a). The additional absorption peak could indicate the presence of an intermediate state that is involved in the energy transfer process leading to the observed emission in the PL spectrum. In the overlay of the PL and PLE spectra in Fig. S5b, two clear resonances are visible at the energies of 2.95 eV (matching free exciton energy) and 2.97 eV.

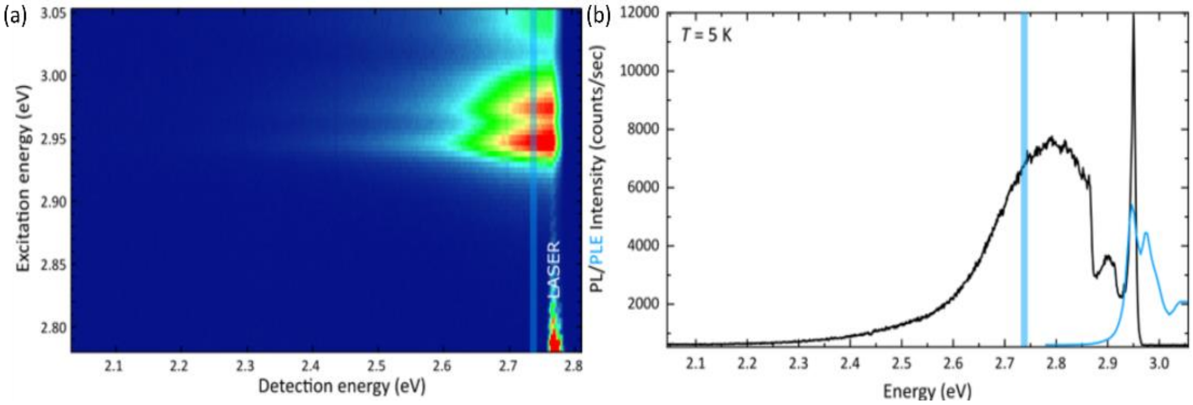

Figure S5. PL and PLE (quasi-absorption) characterization of $CuBr_2$ crystals. (a) False colour PLE intensity map of bulk $CuBr_2$. Strong emission begins to emerge when the excitation energy is close to the free exciton emission energy. (b) A comparative analysis of PL and contour plot of the PLE data. Blue region marks the detection energy.

Figure S6 describes the procedure of the $CuBr_2$ sample preparation for photoluminescence measurement. The $CuBr_2$ flakes are encapsulated between hBN flakes, by dry transfer method. A $CuBr_2$ flake previously exfoliated on PDMS was deposited on the bottom hBN located on the corner of a 90 nm SiO_2/Si substrate. The top hBN was picked up by the PDMS/PPC stack at 40 °C and transferred at 70 °C on top of the $CuBr_2$/bottom hBN stack. These heterostructures with $CuBr_2$ and hBN were mapped using sub-band excitation energy (Fig. S6c-h). Many narrow lines were found on such samples exhibiting emission in the energy range 2.0 – 2.5 eV. When compared to the single particle band gap found from the comparison between the ultraviolet photoemission spectroscopy and tunneling spectroscopy at the energy of about 5 eV, such emitting centers can be attributed to midgap intradefect transitions. The different spectral characteristics of individual emitting centers point towards various origins, such as vacancies, impurities, and/or adsorbed functional groups. This result indicates a great potential for the application of this material to host sources of single photon emitters, especially given the rather good stability of the observed narrow lines in time.

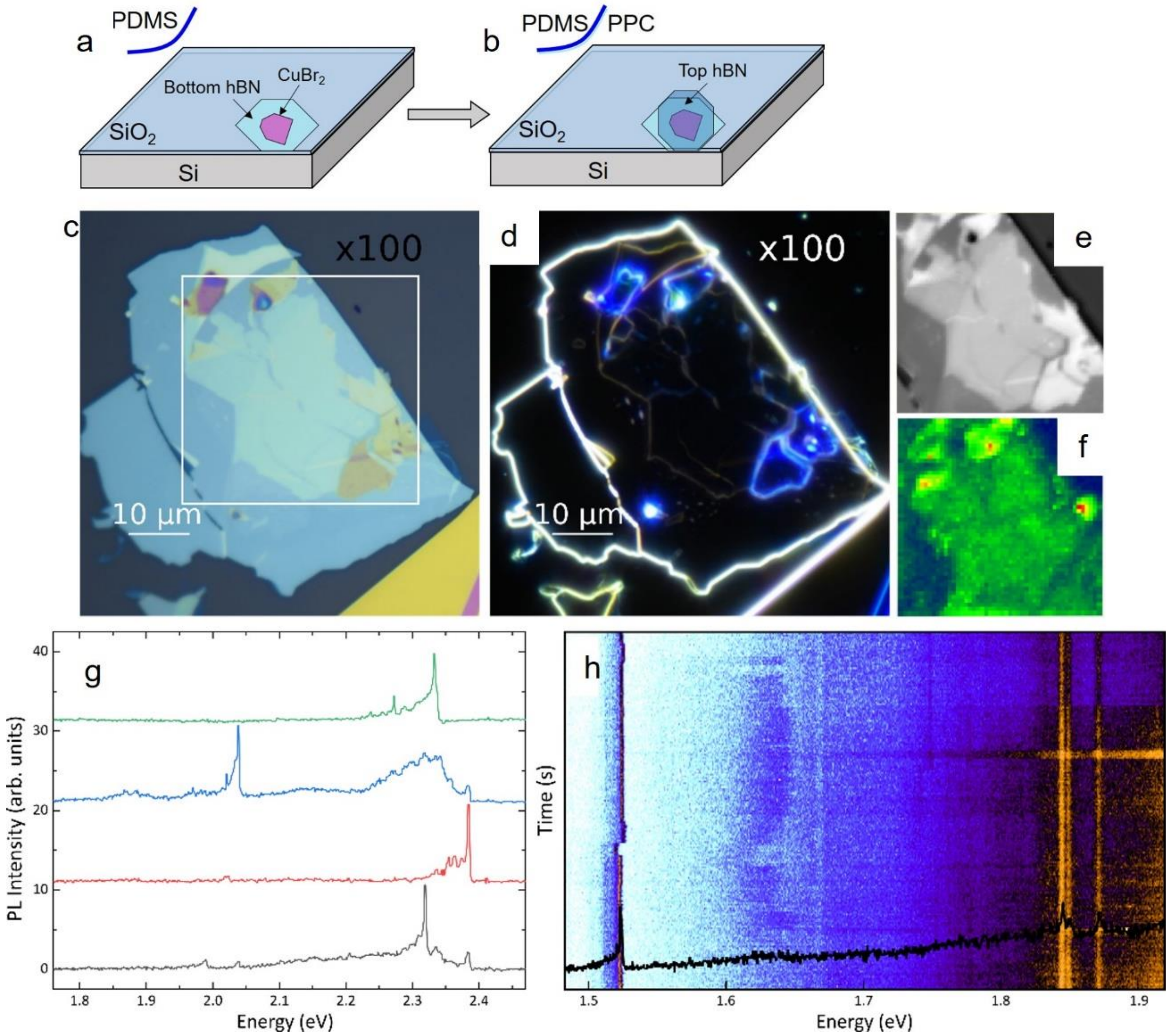

Figure S6. Fabrication process of the $CuBr_2$ samples for PL measurements. (a) Transfer of $CuBr_2$ flakes on bottom hBN flakes on Si/SiO_2 substrate by PDMS stack. (b) Transfer of top hBN on $CuBr_2$/bottom hBN stack by PDMS/PPC stack. (c) Optical image of hBN/$CuBr_2$/hBN heterostructure. (d) Dark field optical microscopy image of the stack shows sharp edges of the flakes and no liquid bubbles, which means no flake degradation and clean interface of the heterostructure. (e) Reflected laser map and (f) emission intensity false colour map of the region indicated with a white square in panel (c). Sample was excited with 2.33 eV laser source at 4.2 K. Bright red spot indicates location of narrow line appearance. (g) Spectra from selected spots showing intense narrow lines against a background of broad emission covering wide range of visible light (1.9 eV to 2.4 eV). (h) Time dependence (over 100 s) of the emission on selected spot indicating good stability of the possible single photon emitter.

4. Micro-ultraviolet photoemission spectroscopy (UPS) measurement

We studied the $CuBr_2$ 2D flake using micro-UPS technique. The $CuBr_2$ was exfoliated on a 90-nm thick SiO_2/Si substrate in a glove box. Figure S7 shows the flakes of interest with a range of thicknesses ranging from 2 to over 40 layers. Thin flakes such as 1 and 2 shown in panel a, offer a faint contrast. Flakes close to 40 layers are in purple colour. The AFM micrographs (Fig. S7 b) of the flake 1 show a thickness of 2.1 nm (Fig. S7 c) that is equivalent to three atomic layers; flake 2 on the other hand, is

around 3.4 nm thick (Fig. S6d), equivalent to 5 layers of $CuBr_2$. A summary of the flakes' height extracted from the AFM study is presented Table S1.

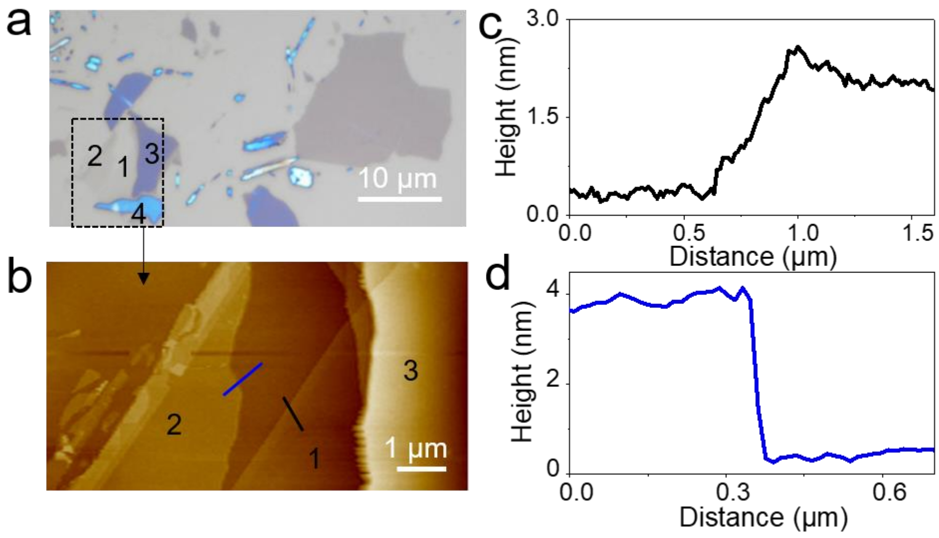

Figure S7. Height profiles of $CuBr_2$ flakes used for micro-ultraviolet photoemission spectroscopy. (a) Optical images of the $CuBr_2$ flakes with a varied thickness exfoliated on a 90-nm thick SiO_2/Si substrate. (b) AFM micrograph of $CuBr_2$ flakes 1 to 3 presented in panel **a**. Flake 1 (panel c) and flake 2 (panel d) height profiles showing 2.1 and 3.4 nm, respectively.

Table S1. AFM height analysis of flakes 1-4 shown in Fig. S7.

Flake No.	Thickness (nm)	Number of layers
1	2.1	3
2	3.4	5
3	25.0	39
4	52.3	82

Figure S8 depicts the flow chart of $CuBr_2$ fabrication for micro-UPS measurement. The exfoliated flakes on the SiO_2/Si substrate (Fig. S8a) were transferred onto a freshly cleaned highly doped Si substrate (Fig. S7b) which enables a good grounding for work function measurement. Pre-patterned metal markers on the Si substrate are designed to help navigation for the UPS measurement on desired flakes. The flakes are covered by a single layer graphene to prevent them from degradation (Fig. S8c). To note, we used mechanically exfoliated monolayer graphene to encapsulate the $CuBr_2$ flake. Integrity and quality of the graphene layer are evaluated by using optical microscopy and Raman spectroscopy. The contact made between the graphene and subsequent deposited gold electrode secures the grounding of the flakes (Fig. S8d).

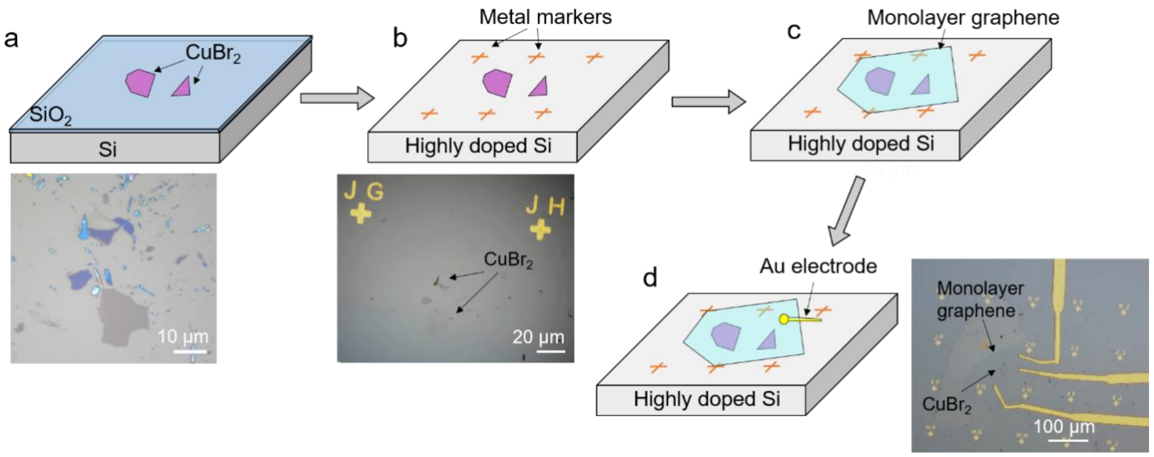

Figure S8. Fabrication process of $CuBr_2$ samples for micro-ultraviolet photoemission spectroscopy measurement. After $CuBr_2$ exfoliation on a Si/SiO_2 substrate (Fig. S7a), the flakes are transferred to a highly doped Si substrate with pre-patterned markers (Fig. S7b). A SLG flake is deposited on top for protection (Fig. Sc). Panel d shows the final state of the device after the electrodes were fabricated.

Micro-UPS measurements using hv = 21.2 eV are extremely surface sensitive, probing the top few surface layers only. The UPS signal of the device illustrated in Fig. S9 is therefore composed primarily of the spectrum of the graphene overlayer, with a small contribution originating from the flakes underneath. Extracted UPS spectra are shown in Fig. S9 for $CuBr_2$ flakes 1 - 4 alongside the spectrum extracted from a region covered in only graphene. The $CuBr_2$ spectra are dominated by the signal from graphene, which must be subtracted to obtain spectra that are representative of the $CuBr_2$ electronic structure. For this process, the graphene spectrum is matched in the valence band region (< 3.6 eV), where all the spectra are almost identical, and then subtracted. The resulting difference spectrum has been filtered using a Savitzky-Golay polynomial filter to suppress noise. In the main manuscript, filtered spectra are shown as dark lines and unfiltered spectra are represented as points.

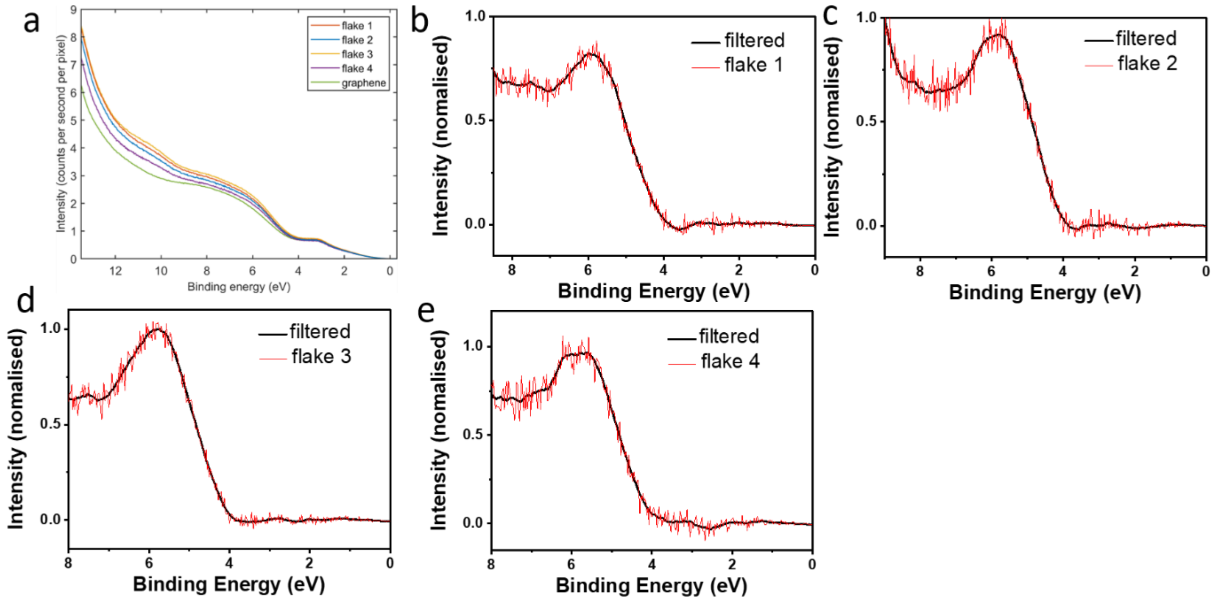

Figure S9. Micro-UPS spectra of (a) $CuBr_2$ flakes 1 to 4 and from a region where only graphene is

present. (b-e) for $CuBr_2$ flakes 1 to4 recorded with a photon energy of 21.2 eV (He I light source) extracted from Figure 3i.

5. $CuBr_2$ device for electronic property measurement

To further investigate the electronic properties of $CuBr_2$, we fabricated a van der Waals (VdW) heterostructure that comprised a few-layer $CuBr_2$ flake (11 nm thick) encapsulated between two single-layer graphene (SLG) flakes, which provided electrical connection to the dihalide flake (Fig. S10a). The resulting layered structure is shown in panel b, where the electrical connection is as well represented schematically. Applying a DC bias across the stack allows us to probe vertical transport, as illustrated by the circuit schematic (panel b). Panel c on the other hand, offers a top view of the four terminal electrical contacts used, which allows us to probe lateral transport through the graphene, using AC. First, we measured room temperature resistance as a function of back gate voltage (V_{BG}). The resulting curves, referred to here as the Dirac curves, are shown in Fig. S10d for two distinct regions of the device: graphene on SiO_2 (black) and graphene on $CuBr_2$ (blue). The Dirac curve for graphene on SiO_2 closely resembles that of pristine SLG, with a sharp and symmetric peak close to zero (V_{BG}), indicating good quality of this SLG flake. The peak is not perfectly centred at 0 V_{BG}, implying the presence of a small amount of electron doping. This is in stark contrast to the Dirac curve for graphene on $CuBr_2$, which shows a much broader peak that is shifted from zero by a greater degree and in the opposite direction, indicating a significant hole doping due to the $CuBr_2$. Dirac curves allow us to extract mobility, μ, and density disorder, n_i, for the distinct SLG regions. To quantitatively analyse the Dirac curves, we fit the resistance (*R)* peaks and thus extracted the mobility of graphene (μ*)*, the voltage at which the peak (neutrality point) occurs (V_D) and the inhomogeneity in carrier density (n_i). The values extracted from our fits are summarised in Table S2.

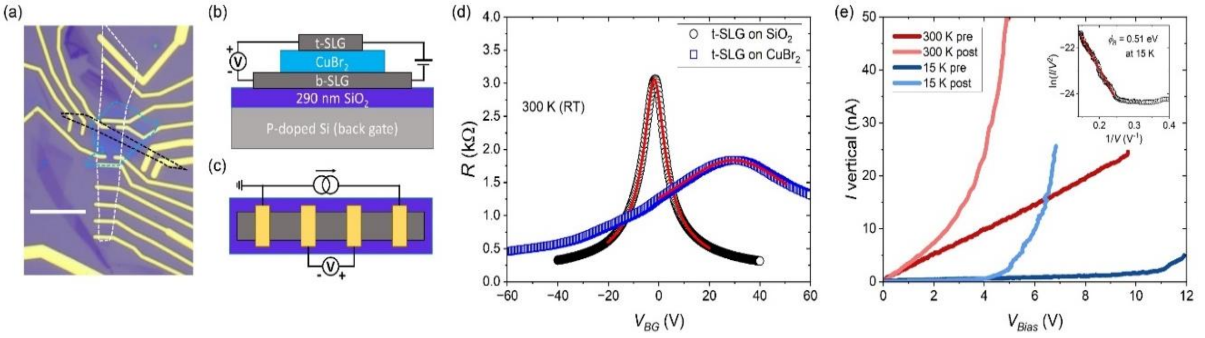

Figure S10. Charge transport in few-layer $CuBr_2$ encapsulated by SLG. (a) Optical micrograph of the device, with top SLG (white contour), few-layer $CuBr_2$ (blue contour), and bottom SLG (black contour) flakes outlined. Scale bar represents 10 µm. (b) Cross sectional schematic of the heterostructure and doped Si substrate (used as a back gate). (c) Bird's eye view schematic of device, showing a strip of graphene with four Au contacts laid on top. (d) Lateral transport in graphene, the 4-terminal resistance measured while varying back gate voltage, for two regions of the top SLG flake: on SiO_2 (black symbols) vs on $CuBr_2$ (blue symbols). Red lines show Dirac curve fits. Measurements are taken at room temperature (300 K). (e) Vertical transport through $CuBr_2$. Current through the stack measured while DC bias voltage between the top and bottom graphene flakes is increased. At both high (300 K) and low (15 K) temperature, the $CuBr_2$ underwent dielectric breakdown for V > 12 V and remained in a

more conductive state; *I-V* curves are shown for before and after this transition. Inset shows $\ln(I/V^2)$ against $1/V$, for low temperature "post" breakdown *I-V* data. Red line is a linear fit to the data, which allows us to estimate a potential barrier height at the top-SLG/$CuBr_2$ interface of 0.51 eV.

Table S2. Summary of transport parameters and their associated errors (δ) extracted from fits to Dirac curves for Gr/SiO_2 and Gr/$CuBr_2$ regions, respectively.

Region	V_D (V)	δV_D (V)	μ ($cm^2 V^{-1} s^{-1}$)	$\delta\mu$ ($cm^2 V^{-1} s^{-1}$)	n_i (cm^{-2})	δn_i (cm^{-2})
Gr/SiO_2	-1.80	0.02	6610	21	$0.27\times \mathbf{10^{12}}$	$1\times \mathbf{10^{9}}$
Gr/$CuBr_2$	29.63	0.02	1810	3	$2.09\times \mathbf{10^{12}}$	$3\times \mathbf{10^{9}}$

The graphene on $CuBr_2$ has a lower mobility and a higher induced carrier density inhomogeneity than the pristine case (on SiO_2), which implies a high level of charge transfer occurs at the interface between SLG and $CuBr_2$. The latter is confirmed by the relative shift in the position of the Dirac peak, where graphene on $CuBr_2$ shows a large degree of hole doping (and consequently graphene dopes $CuBr_2$ with electrons). Equivalent analysis conducted for the bottom-SLG flake shows similar charge transfer effects in the region contacting the $CuBr_2$.

Next, we probed vertical transport through the stack. Room temperature (300 K) and low temperature (15 K) *I-V* curves for the $CuBr_2$ flake are shown in Fig. S10e. In both cases, we found that the $CuBr_2$ was initially in an insulating state (plots labelled as "pre"), but as we increased the bias, the dielectric break down took place and more conduction through the flake became possible (plots labelled as "post"). The low-bias resistance of the $CuBr_2$ came to R_{RT} = 414 MΩ and R_{LT} = 8.64 GΩ for 300 K and 15 K, respectively. The large increase in resistance at low temperature is attributed to a reduction in conduction due to thermally excited carriers over the energy barrier formed at the Gr/$CuBr_2$ interface. After breakdown, the *I-V* curves became strongly non-linear in the conductive state. For the 15K data, this non-linear behaviour implies that vertical transport through the $CuBr_2$ occurs in the Fowler-Nordheim tunnelling regime[2]. The inset of Fig. S10e shows the non-linear ("post" breakdown) data for 15 K, plotted as $\ln(I^2/V)$ against $1/V$. We see a linear dependence for *I-V* data above 4 V, which confirms the tunnelling nature of transport. In this regime we can fit a linear slope to our $\ln(I^2/V)$ data and estimate the height of the barrier, using the equation

$$\ln\left(\frac{I}{V^2}\right) \sim -\frac{8\pi\sqrt{2m^*}\varphi_B^{3/2} d}{3heV},$$

where, φ_B is the barrier height, d is the barrier thickness, m^* is the effective mass, and h is Planck's constant. We make the approximation that m^*~the free electron mass and extract a barrier height $\varphi_B \sim 0.51$ eV. A similar analysis on the non-linear part of the "pre" breakdown data, above 10 V, yields a barrier height of $\varphi_B \sim 1.10$ eV. Hence, applying such a high bias across the $CuBr_2$ flake has altered its electronic properties and lowering the potential barrier formed at the interface with graphene. Considering this barrier would form a fraction of the total bandgap of the material, our extracted values are congruent with the DFT calculations, shown in Fig. 4, which predict a wide (>1 eV) band gap for few layer $CuBr_2$. This is interesting, as density of states calculations for bulk $CuBr_2$ predict it to show metallic behaviour[3].

6. Characterization of bulk and thin flake of metal dihalides (MX_2, M=Cu, Co, Ni, X=Cl, Br, I)

Figure S11 shows few example measurements of X-ray diffraction (XRD) of MX_2 ($CuCl_2$, $CoCl_2$, $NiBr_2$, CoI_2). Due to the sensitivity of samples to the air, vacuum bags and hermetic cell were used during the XRD characterization. O-rings were used to seal a Kapton polymide film (Dupont pty ltd) to allow the X-ray to transmit through while protecting the samples from the moisture outside.

The observed peaks are well fitted with the standard referenced data. For $CuCl_2$, it belongs to C2/m space, with a = 6.9 Å, b = 3.3 Å, c = 6.8 Å and β = 122.2°. [4] $CoCl_2$ is from R32 space group, with a = 3.5 Å, b = 3.5 Å, c = 17.4 Å, and β = 90°. [5] $NiBr_2$ is from $R\bar{3}m$ space group as well, with a = 3.7 Å, b = 3.7 Å, c = 18.3 Å and β = 90°.[6] CoI_2 is from P-3m1 space group, with a = 4.0 Å, b = 4.0 Å, c = 6.7 Å, and β = 90°.[6, 7] Apart from the characteristic peaks, the presence of additional peaks (Fig. S11) reveals that metal dihalides degraded to some extent due to the moisture.

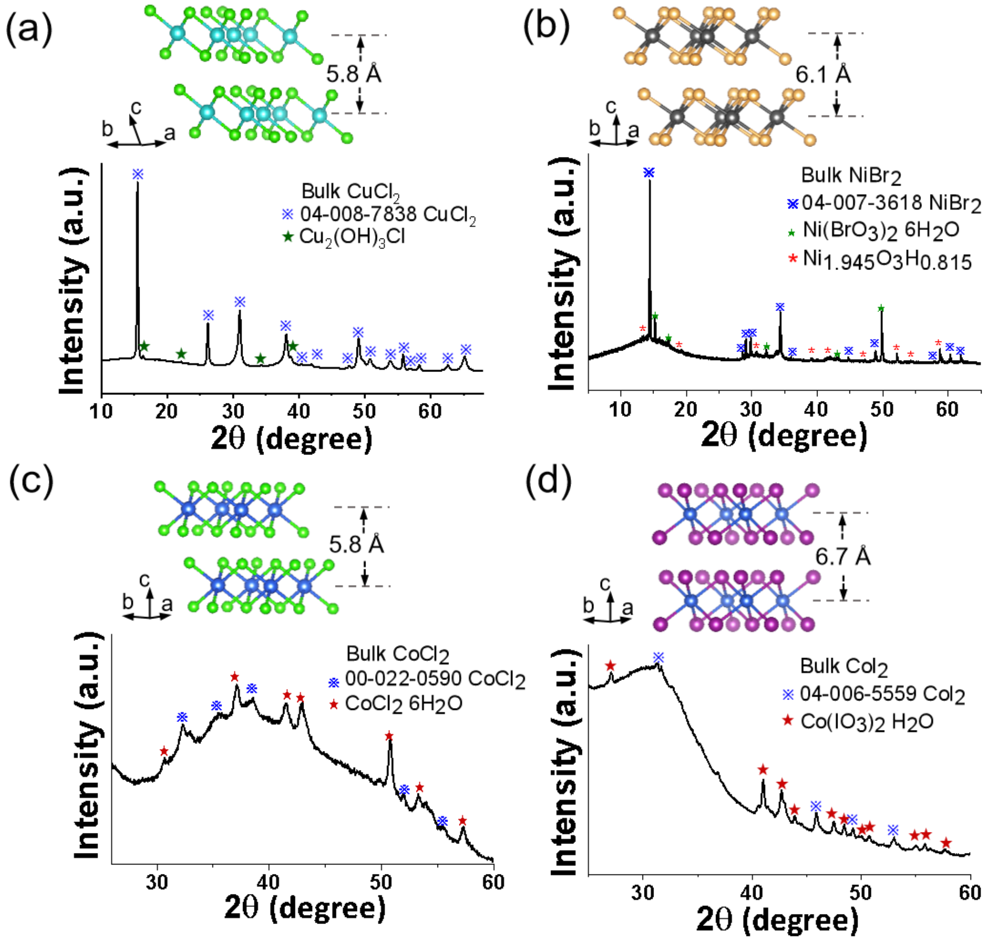

Figure S11. (a-d) Top images: schematic view of the layered structure of metal dihalides: $CuCl_2$, $NiBr_2$, $CoCl_2$ and CoI_2 respectively. Below are powder XRD patterns accordingly. $NiBr_2$ is measured using Agilent Supernova while $CuCl_2$, $CoCl_2$, CoI_2 are measured using a Rigaku tool. Cu atom is in cyan colour, Br atom is in orange colour, Cl atom is in green colour, Ni atom is in grey colour, Co atom is in blue colour, I atom is in purple colour.

To further characterize the exfoliated 2D metal halides, we used the same exfoliation method and carried out Raman spectroscopy, the spectra of the bulk and 2D flakes of MX_2 are shown in Fig. S12. The exfoliation process was done in a glovebox, and the corresponding optical images, and AFM profiles of flakes appear in Fig. S12. All the Raman spectra are recorded on the samples protected by a hermetic cell. Please see more details about the hermetic cell including its schematic set-up and digital photograph in Section 3 below. For bulk and 2D $CoCl_2$, E_g mode (Fig. S12a) occurs at ~160 cm^{-1} [8]. $NiBr_2$'s E_g and A_g position (Fig. S12c) at 110 cm^{-1} and 175 cm^{-1}, respectively. [9] For CoI_2, E_g and A_g (Fig. S12b) are shown at 130 cm^{-1} and 230 cm^{-1} [10].

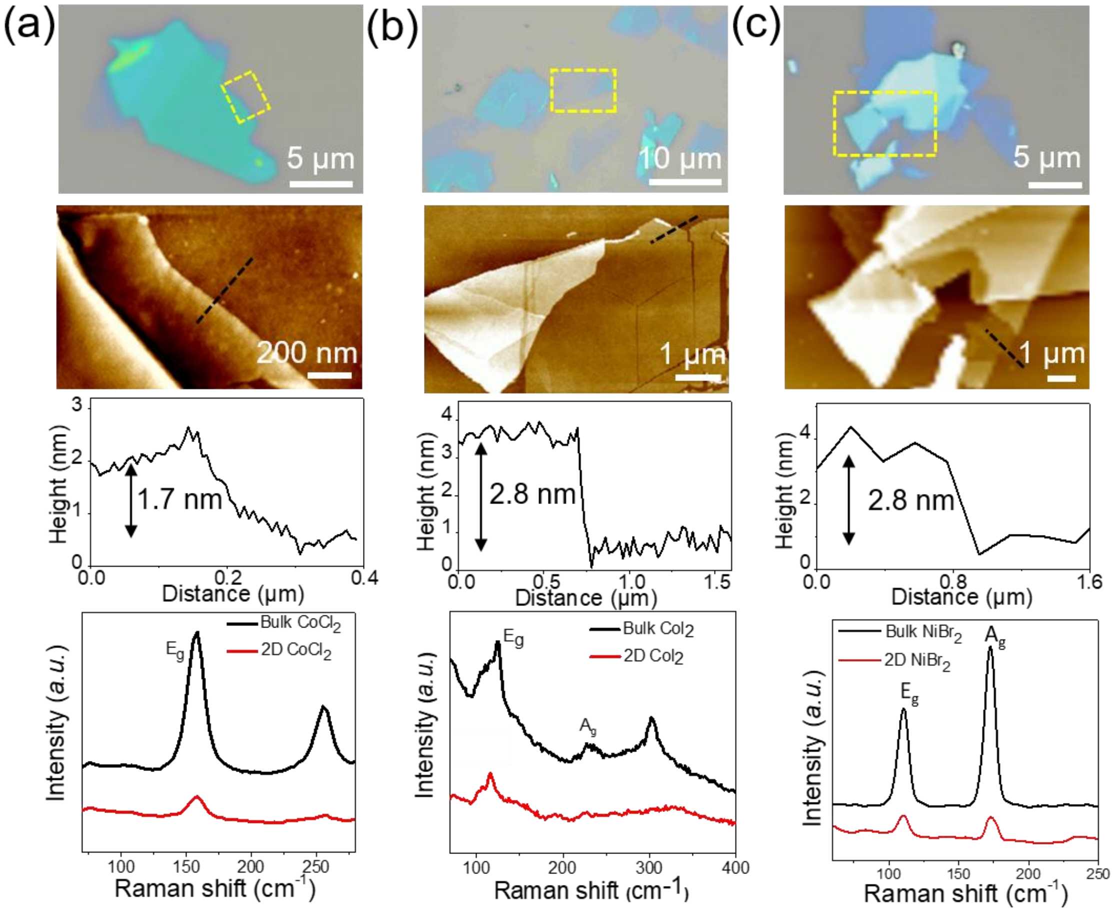

Figure S12. Optical images and Raman spectra of the as-recrystallized and exfoliated metal- (Co, Ni) bromide, chloride and iodide. The flakes are not degraded when being kept within an inert environment . (a) $CoCl_2$, (b) CoI_2, (c) $NiBr_2$.

7. Stability tests of 2D flakes

We conducted the stability test and compared the flakes made by different exfoliation conditions and stored in either a glovebox or kept in air. Fig. S13 illustrates the schematic of a hermetic cell (HC) that was used to keep the sample from degradation.[11] Then, an initial Raman characterisation of the sample was performed. The HC containing the sample was then taken out from the glovebox and left in air for over 30 mins. The $CuBr_2$ flakes did not show any signs of degradation, while retaining their

smooth surface. However, as soon as the sample was taken out of the HC, the surface of the flakes starts degrading such that the flake edges are not sharp anymore and the flake surface becomes rough. In another experiment, we exfoliated the flakes in air and Raman was done in air. As a comparison, we show the optical images of $CuBr_2$ (Fig. S14) prepared as follows: exfoliated in glovebox and tested in hermetic cell, exfoliated in glovebox and tested in air, exfoliated in air and tested in air respectively, and measured them in two different ranges, before 400 cm^{-1} and 3000-4000 cm^{-1}. The flakes exfoliated in glovebox and measured in HC are in the best condition without any degradation and show A_g, B_g and A_g Raman modes (seen in Fig. S14f) with no sign of water peak at 3400 cm^{-1} (given in Fig. S14i). However, once the flakes were exposed to air either in exfoliation procedure or during measurement, material degradation becomes obvious, which is consistent with the results shown in Fig. S14d & S14e. Meanwhile a strong 3400 cm^{-1} peak appears, indicating the presence of water.

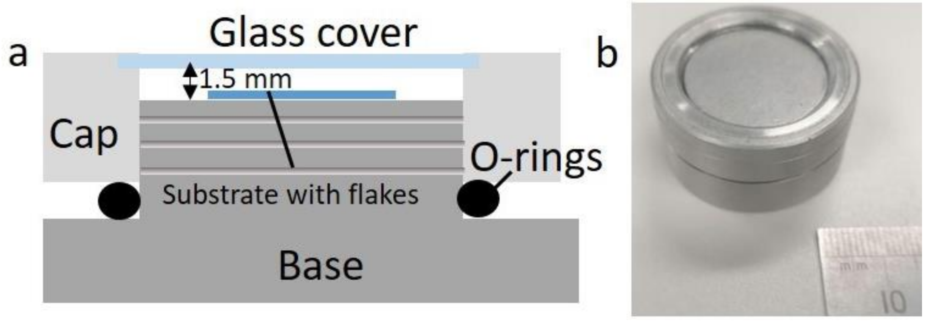

Figure S13. Hermetic cell for sample preservation and analysis. Simple schematic of the cell showing the sample location and optical access for optical characterization (a). Panel b showing a picture of the real encapsulation cell. The ruler on the bottom provides a real-size reference.

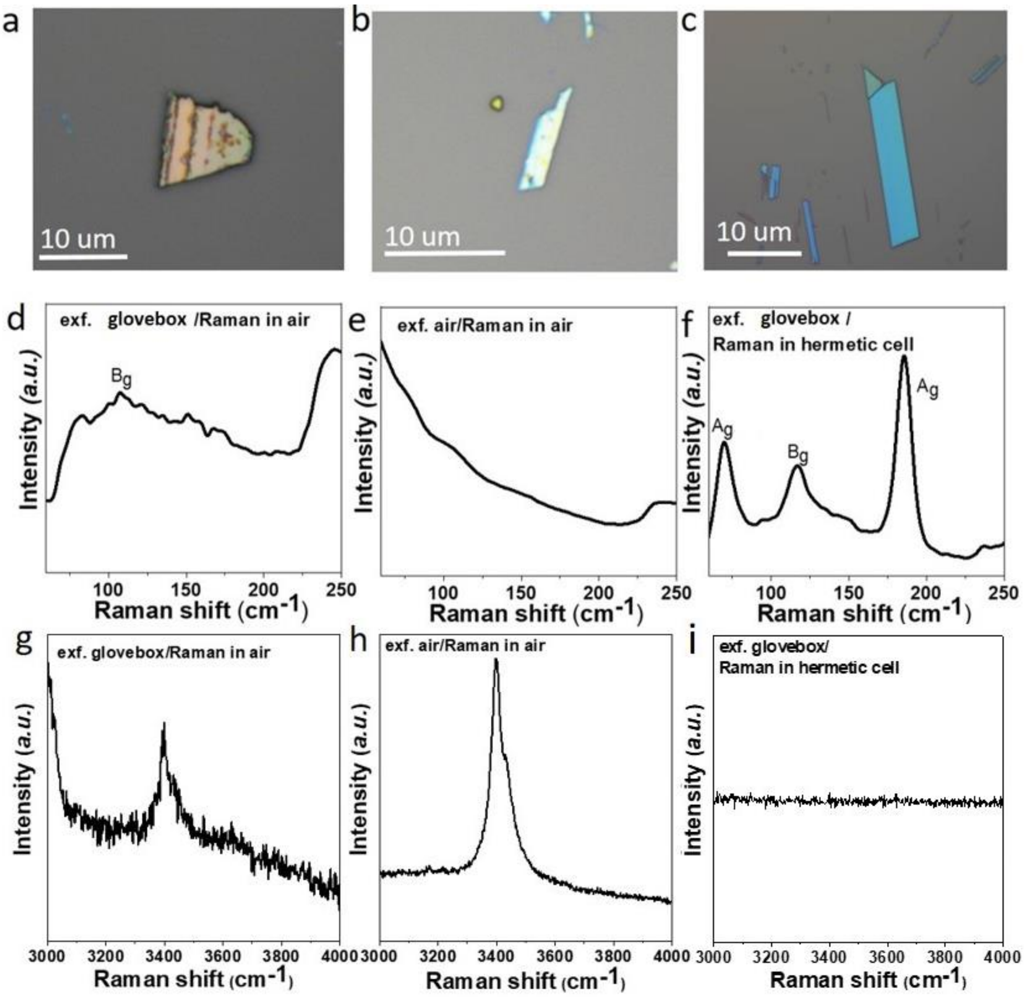

Figure S14. Stability test of $CuBr_2$. Optical images of $CuBr_2$ exfoliated in glovebox inside an inert atmosphere but Raman-analysed in air (a); exfoliated and Raman-measured in air (b): exfoliated in glovebox and Raman-measured inside the hermetic cell (c). Raman analysis from the corresponding flakes shown in panels a to c, from 50 to 400 cm^{-1} (d to f) and from 3000 to 4000 cm^{-1}.

To test the encapsulation by graphene versus hBN for the stability, we encapsulated mechanically exfoliated thin flakes of NiI_2 crystals using hBN and graphene and measured the Raman spectra at various time intervals. As comparison, we also measured the bare NiI_2 thin flakes (i.e. without any encapsulation). All the NiI_2 flakes were mechanically exfoliated on SiO_2 (290 ± 10 nm)/Si wafers inside N_2 filled glovebox and encapsulation with hBN and graphene was also carried out inside the glovebox. We compare the characteristic peaks assigned to E_g and A_g of NiI_2. In the case of both hBN-NiI_2 and graphene-NiI_2, Raman spectra recorded at 5 min, 10 min, 1 hr and 1 day did not show any significant changes and characteristic peaks remain at expected position for E_g and A_g of NiI_2. The encapsulation of metal halides with hBN and graphene is stabilizing thin crystals from degradation. In contrast, E_g and A_g peaks of NiI_2 flake without encapsulation (bare-NiI_2) disappeared soon after exposure to ambient air within 5 mins. The flake degradation could proceed by dissolution, oxidation, or swelling of the material and the kinetics might be difficult to follow as highlighted in reference[12].

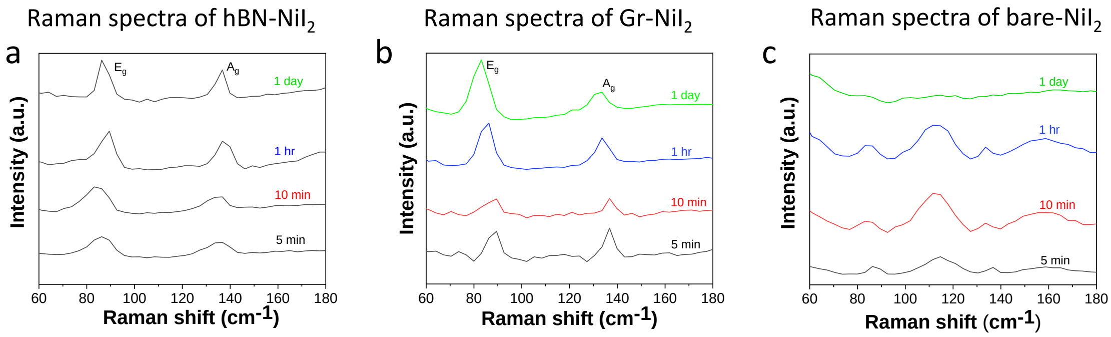

Figure S15. Raman spectra of freshly exfoliated NiI_2 flake inside the glovebox and encapsulated with (a) hBN, (b) graphene and (c) without any encapsulation, with measurement done in ambient atmosphere at various time intervals. Flakes are exposed to ambient atmosphere as soon as they are taken out from the glovebox and measured the Raman spectra are measured at four time intervals (5 mins, 10 mins, 1 hr and 1 day) with continuous exposure to ambient conditions.

Below, we show complete set of images pertaining to Figure 6d, from the *in situ* AFM imaging while testing the stability of the metal-halide flakes. The starting RH was around 9.2 % RH when glovebox mounted sample was transferred to AFM. To adjust the RH, the N_2 gas flow was reduced to allow the air into the sample holder. When the N_2 flow was completely stopped, the RH stabilized around 15-20%. To further increase the RH, we introduced atmospheric air in controlled volume into the sample chamber using a syringe.

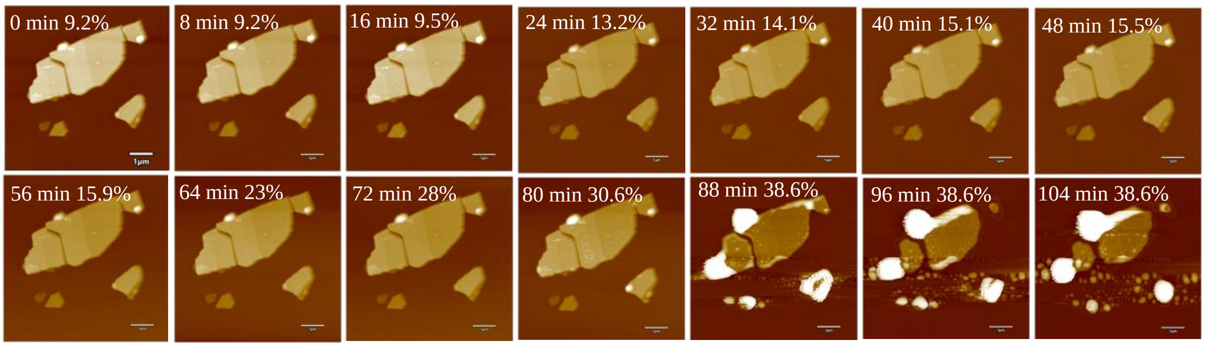

Figure S16. In-situ AFM imaging of the NiI_2 flakes' degradation process from 9.2% relative humidity (RH) to 38.6% RH. The surface of the targeted NiI_2 is smooth at the low RH, began to be rough at around 30% RH. Later, it starts to absorb more moisture until parts of the flakes disappear at higher RH. Each image took 8 minutes, and the RH was modulated continuously as indicated on the images, along with the timepoint at which the image was taken.

8. Optical microscopy of 2D $CuCl_2$

To establish a robust and quick identification method for characterizing the metal halide flakes, we simulated the contrast plot over several thicknesses of $CuCl_2$ flake using the Fresnel equation and compared it with the experimental result. As we exfoliated the $CuCl_2$ flakes on 90 nm SiO_2/Si substrates, the contrast value depends on the intensities of both flakes and substrates under filters (seen in Fig.

S17a). The intensities of flake and substrate are calculated by Fresnel equations, as shown in equation 1 and 2. Here, *d* is the thickness of SiO_2, λ is the wavelength of light under filters. The subscripts 0, 1, 2 and 3 refer to air, $CuCl_2$, SiO_2 and Si respectively as given in Fig. S17a. The r_{jk} refer to the reflected amplitude at the interface of two media *j* and *k*. Φ_j is the phase shift depending on the complex refractive index and thickness of the media *j*, as well as the illumination wavelength. The optical intensity *C* of the flake is extracted over the substrate and flake. Optical contrast is obtained from the Michelson contrast (Equation 5). We selected a 500 nm longpass optical filter as it provides a broad contrast value across a wide range of flake thicknesses, which enlarges the datasets for a better prediction (see the contrast map in Fig. S17b).

Regarding the simulated data, we follow the equations 1 to 6 to compute $CuCl_2$ contrast at proposed conditions. *n* is the refractive index, defined by the ratio of the speed of light in the first and the second medium. *k* is the extinction coefficient. Both *n* and *k* are derived from the ellipsometry measurement result of the bulk $CuCl_2$ powders, shown in Fig. S17c. Following the same described methodology [13], we modelled the correlation of the optical contrast with the $CuCl_2$ thickness, which is given as the red line of Fig. 5e.

$$I_{substrate}(\lambda) = \left| \frac{r_{02} + r_{23}e^{-2i\Phi 2}}{1+r_{02}r_{23}e^{-2i\Phi 2}} \right|^2 \quad (1)$$

$$I_{flake}(\lambda) = \left| \frac{r_{02}e^{i(\Phi 1+\Phi 2)} + r_{12}e^{-i(\Phi 1-\Phi 2)} + r_{23}e^{-i(\Phi 1+\Phi 2)} + r_{01}r_{12}r_{23}e^{-i(\Phi 1+\Phi 2)}}{e^{i(\Phi 1+\Phi 2)} + r_{01}r_{12}e^{-i(\Phi 1-\Phi 2)} + r_{01}r_{23}e^{-i(\Phi 1+\Phi 2)} + r_{12}r_{23}e^{i(\Phi 1-\Phi 2)}} \right|^2 \quad (2)$$

$$r_{jk} = \frac{\tilde{n}_j - \tilde{n}_k}{\tilde{n}_j + \tilde{n}_k} \quad (3)$$

$$F_j = \frac{2p\tilde{n}_j d_j}{l} \quad (4)$$

$$C(d, \lambda) = \frac{I_{flake} - I_{substrate}}{I_{flake} + I_{substrate}} \quad (5)$$

$$\tilde{n} = n - ik \quad (6)$$

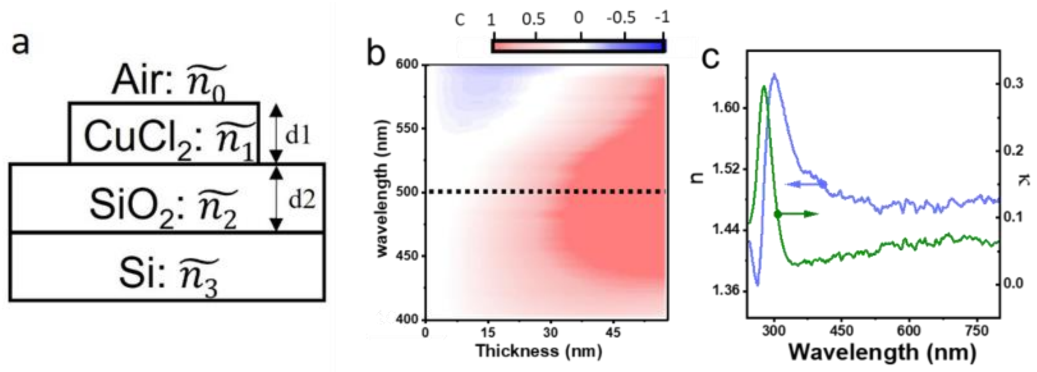

Figure S17. Fresnel model for optical contrast of $CuCl_2$ flakes. (a) The $CuCl_2$ sample on the substrate with different thicknesses and refractive indices labelled. (b) Colormap which shows the dependence of optical contrast *C* with illumination wavelength and $CuCl_2$ thickness. (c) *n* and *k* data collected from ellipsometry.

References

(1) Wang, C.; Yu, D.; Liu, X.; Chen, R.; Du, X.; Hu, B.; Wang, L.; Iida, K.; Kamazawa, K.; Wakimoto, S.; et al. Observation of Magnetoelastic Effects in a Quasi-One-Dimensional Spiral Magnet. *Phys. Rev. B* **2017**, *96*, 085111, DOI: 10.1103/PhysRevB.96.085111.

(2) Wang, Z.; Gutiérrez-Lezama, I.; Ubrig, N.; Kroner, M.; Gibertini, M.; Taniguchi, T.; Watanabe, K.; Imamoğlu, A.; Giannini, E.; Morpurgo, A. F. Very Large Tunneling Magnetoresistance in Layered Magnetic Semiconductor Cri3. *Nat. Comm.* **2018**, *9*, 2516, DOI: 10.1038/s41467-018-04953-8.

(3) Altarawneh, M.; Marashdeh, A.; Dlugogorski, B. Z. Structures, Electronic Properties and Stability Phase Diagrams for Copper(I/Ii) Bromide Surfaces. *Phys. Chem. Chem. Phys.* **2015**, *17*, 9341-9351, DOI: 10.1039/C4CP05840B.

(4) Burns, P. C.; Hawthorne, F. C. Tolbachite, $Cucl_2$, the First Example of Cu^{2+} Octahedrally Coordinated by Cl^{-}. *Am. Mineral.* **1993**, *78*, 187-189.

(5) Brusset, H.; Gillier-Pandraud, H.; Bkouche-Waksman, I. Structure Comparée Du Composé $Cocl_2$, $4ch_3oh$ Et Des Solvates D'halogénures De Métaux Divalents. *Bull. Minéral.* **1968**, *91*, 549-556.

(6) Y. Tokunaga; D. Okuyama; T. Kurumaji; T. Arima; H. Nakao; Y. Murakami; Y. Taguchi; Tokura, Y. Multiferroicity in $Nibr_2$ with Long-Wavelength Cycloidal Spin Structure on a Triangular Lattice. *Phys. Rev. B* **2011**, *84*, 060406(R), DOI: 10.1103/PhysRevB.84.060406.

(7) Silva, A. J. R. d.; Falicov, L. M. Calculation of Optical Transitions in Nii_2 and Coi_2 under Pressure. *Phys. Rev. B* **1992**, *45*, 11511-11517, DOI: 10.1103/PhysRevB.45.11511.

(8) D. J. Lockwood; I.W. Johnstone; Mischler, G.; Carrara, P. Raman Scattering from Magnons in $Cocl_2$ and $Fecl_2$. *Solid State Commun.* **1978**, *25*, 565—568, DOI: 10.1016/0038-1098(78)91490-4.

(9) White, M. A.; Chieh, C.; Anderson, A.; Staveley, L. A. K. Calorimetric, Spectroscopic, and Structural Studies of Anhydrous Zinc Bromide. *J. Chem. Phys.* **1984**, *80*, 1254, DOI: 10.1063/1.446803.

(10) Mischler, G.; Lockwood, D. J.; Zwick, A. Raman Scattering from Electronic Excitations and Phonons in Coi_2. *J. Phys. C: Solid State Phys.* **1987**, *20*, 299-309, DOI: 10.1088/0022-3719/20/2/012.

(11) Thompson, J. P.; Doha, M. H.; Murphy, P.; Hu, J.; Churchill, H. O. H. Exfoliation and Analysis of Large-Area, Air-Sensitive Two-Dimensional Materials. *J. Vis. Exp.* **2019**, *143*, e58693, DOI: 10.3791/58693.

(12) Zhang, T.; Grzeszczyk, M.; Li, J.; Yu, W.; Xu, H.; He, P.; Yang, L.; Qiu, Z.; Lin, H.; Yang, H.; et al. Degradation Chemistry and Kinetic Stabilization of Magnetic Cri_3. *J. Amer. Chem. Soc.* **2022**, *144*, 5295-5303, DOI: 10.1021/jacs.1c08906.

(13) Skaar, J. Fresnel Equations and the Refractive Index of Active Media. *Phys. Rev. E* **2006**, *73*, 026605, DOI: 10.1103/PhysRevE.73.026605.
